# Mind–body exercise for improving balance, aerobic capacity, walking ability, muscle strength, and mental health in patients with type 2 diabetes: a network meta-analysis

**DOI:** 10.3389/fendo.2026.1749651

**Published:** 2026-03-04

**Authors:** Fuwei Wu, Yanling Yuan, Yingchun Hou, Zhen Ye

**Affiliations:** 1 Graduate School of Physical Education, Myongji University, Yongin, Republic of Korea; 2 School of Physical Education, Shandong Sport University, Jinan, Shandong, China; 3 School of Physical Education, University of Jinan, Jinan, Shandong, China; 4 Department of Chinese Medicine, Shangrao Guangxin District People’s Hospital, Shangrao, Jiangxi, China

**Keywords:** mind–body exercise, Network meta-analysis, pilates, qigong, Tai chi, type 2 diabetes mellitus, yoga

## Abstract

**Objective:**

To compare and rank the effects of different mind–body exercise interventions on psychological and physical outcomes in individuals with type 2 diabetes mellitus using a network meta-analysis.

**Methods:**

This study systematically searched PubMed, Embase, Web of Science, and the Cochrane Library, and conducted a frequentist network meta-analysis using Stata with SUCRA-based ranking; risk of bias was assessed using RoB 2.0, certainty of evidence evaluated with CINeMA, and robustness examined through sensitivity analyses and publication bias assessment using funnel plots.

**Results:**

This study provides preliminary, outcome-specific evidence based on 13 randomized controlled trials involving over 500 individuals with type 2 diabetes mellitus. Network meta-analysis suggested that Pilates significantly improved balance ability (SMD = 1.52, 95% CI 0.77–2.28) and walking ability (SMD = 1.20, 95% CI 0.28–2.12) compared with control. Walking Meditation showed a favorable effect on aerobic capacity (SMD = 1.40, 95% CI 0.44–2.37), while yoga improved muscle strength (SMD = 0.79, 95% CI 0.26–1.33). Mindfulness-based interventions were associated with reduced anxiety (SMD = 1.05, 95% CI 0.46–1.63). Sensitivity analyses indicated that physical function outcomes were generally robust, whereas psychological outcomes were sensitive to influential studies. The certainty of evidence was predominantly very low according to CINeMA assessment.

**Conclusion:**

This network meta-analysis provides preliminary, outcome-specific evidence that mind–body exercise may improve physical function in type 2 diabetes, while effects on psychological outcomes remain uncertain and warrant further high-quality research.

**Systematic Review Registration:**

https://www.crd.york.ac.uk/prospero/, identifier CRD420251133289.

## 1 Introduction

Type 2 diabetes mellitus (T2DM) is one of the most prevalent metabolic disorders worldwide, characterized by insulin resistance and impaired β-cell function, which result in persistent hyperglycemia and multisystem damage (1, 2). With global population aging and ongoing lifestyle transitions, the global prevalence of type 2 diabetes mellitus (T2DM) continues to rise, and the number of affected individuals is projected to reach approximately 1.3 billion by 2050 (3). Beyond metabolic abnormalities, individuals with T2DM frequently experience psychological comorbidities, such as depression, anxiety, and impaired stress perception (4, 5), as well as declines in physical function, including impaired balance, gait disturbances, and reduced muscle strength (6, 7). These complications not only elevate the risk of falls and accelerate functional decline but also substantially reduce quality of life and are strongly associated with poor long-term outcomes (8, 9). Consequently, the development of integrated interventions that simultaneously target both physical function and psychological well-being has emerged as a critical need in the comprehensive management of T2DM.

Exercise interventions are well established as a cornerstone of non-pharmacological management for T2DM, promoting metabolic homeostasis by enhancing insulin sensitivity, improving aerobic capacity, and increasing muscular strength (10). However, conventional exercise programs have primarily focused on glycemic control and cardiopulmonary fitness, while their direct benefits on psychological health remain relatively limited (11). In recent years, mind–body exercises—such as Pilates, yoga, Tai chi, qigong, mindfulness training, and laughter yoga—have gained increasing attention (12). These interventions integrate physical activity with breathing regulation and psychological practice, offering a holistic approach that not only improves balance, gait, and muscular strength but also exerts favorable effects on mental health (13). Potential mechanisms include modulation of the hypothalamic–pituitary–adrenal (HPA) axis, enhancement of autonomic nervous system function, and attenuation of systemic inflammation, collectively contributing to reductions in depression, anxiety, and stress (14). This dual impact on both physical and psychological outcomes highlights the unique value of mind–body exercise in the comprehensive care of individuals with T2DM (15).

Emerging evidence has preliminarily supported the clinical efficacy of mind–body exercise in T2DM. For example, a randomized controlled trial by Yu et al. demonstrated that Pilates significantly enhanced lower-limb strength and improved postural stability (16). Yoga has been shown to increase muscular strength and flexibility, improve respiratory and cardiovascular function, alleviate stress, anxiety, depression, and chronic pain, and further promote sleep quality and overall well-being (17). A 24-week Tai chi program, as well as Tai chi combined with resistance-band training, effectively improved pulmonary diffusing capacity and glycemic control in patients with T2DM (18). Similarly, mindfulness training yielded notable reductions in anxiety symptoms (19). In addition, a meta-analysis indicated that supervised multicomponent exercise performed at least three times per week, with sessions lasting <60 minutes and a cumulative duration ≥180 minutes, represents an evidence-based strategy for improving dynamic balance in individuals with T2DM (20). Another study found that, among older adults with T2DM and mild cognitive impairment, Tai chi was superior to fitness walking or usual care in enhancing balance and preventing falls (21). Nevertheless, most existing studies remain limited to single modalities of mind–body exercise or single outcome domains, and few have systematically compared different interventions within a unified analytical framework. This evidence gap constrains the ability to provide robust, comparative guidance for tailoring individualized exercise prescriptions in clinical practice.

Therefore, this study employed a systematic review and network meta-analysis of randomized controlled trials to comprehensively compare the effects of various mind–body exercise interventions on psychological outcomes (depression, anxiety, and stress) and physical functions (balance, walking ability, muscular strength, and aerobic capacity) in individuals with T2DM. By integrating the existing evidence and ranking interventions through surface under the cumulative ranking curve (SUCRA), this study aimed to delineate the differential benefits of distinct mind–body modalities, identify the most effective strategies, and thereby provide robust evidence to guide individualized rehabilitation, inform updates to clinical practice guidelines, and support public health policy development in the management of T2DM.

## 2 Materials and methods

### 2.1 Study design and protocol registration

This systematic review and network meta-analysis of RCTs followed PRISMA 2020 and its NMA extension. The protocol was registered in PROSPERO (CRD420251133289), and all procedures adhered to Cochrane Handbook standards (22).

### 2.2 Literature search

We systematically searched four major electronic databases—PubMed, Embase, Web of Science, and the Cochrane Library—from their inception to August 7, 2025. The search strategy combined controlled vocabulary (MeSH/Emtree) and free-text terms related to type 2 diabetes mellitus, mind–body exercise (e.g., Yoga, Tai chi, Qigong, Pilates, and Mindfulness-based interventions), and randomized controlled trials, and was adapted to each database. The search was limited to English-language publications, as these databases predominantly index peer-reviewed biomedical literature in English and the majority of randomized controlled trials in this field are published in English. Trial registries and grey literature were not systematically searched to ensure data completeness and methodological consistency for quantitative synthesis; however, reference lists of relevant reviews and included studies were manually screened to minimize the risk of missing eligible trials. A total of 2,809 records were identified and managed using EndNote software. The full search strategies are provided in **Supplementary 1A–D**.

### 2.3 Inclusion and exclusion criteria

Studies were included if they met the PICOS framework: patients diagnosed with type 2 diabetes mellitus (T2DM) without restrictions on age, sex, disease duration, or comorbidities; interventions involving at least one mind–body exercise (e.g., Pilates, Yoga, Tai chi, Qigong, Mindfulness-based interventions, Biofeedback-Assisted Relaxation Training, Guided Imagery Training); comparators such as usual care, diabetes education, lifestyle modification (defined as non–exercise-based interventions focusing on dietary advice, diabetes education, or general lifestyle counseling, and explicitly excluding any structured mind–body exercise components), waitlist control, or other non–mind–body approaches; outcomes reporting at least one psychological (depression, anxiety, stress) or physical function measure (balance, walking ability, muscular strength, aerobic capacity) with sufficient data for effect size calculation; and randomized controlled trials (RCTs) published in English.

Exclusion criteria were non-randomized designs (e.g., cohort, case–control, pre–post studies); studies not specific to T2DM or with mixed populations lacking separate T2DM data; interventions outside the scope of mind–body exercise (e.g., pharmacological, dietary, conventional aerobic/resistance training); studies without relevant outcomes or adequate data; and conference abstracts, case reports, reviews, protocols, or duplicate publications.

### 2.4 Data extraction

Two reviewers independently screened the literature and extracted data, with discrepancies resolved through discussion. During screening, titles and abstracts were first reviewed to exclude irrelevant studies, followed by full-text assessment to determine eligibility. Extracted information included the first author, country and year of publication, participants’ baseline characteristics, sample sizes of intervention and control groups, intervention frequency and duration, details of the intervention protocols, outcome measures, and corresponding results. All references were managed using EndNote X9, and the full texts of eligible studies were retrieved for data extraction. Data were then organized systematically to ensure accuracy and completeness.

### 2.5 Risk of bias assessment

The risk of bias of the included studies was assessed using the Cochrane Risk of Bias 2.0 (RoB 2.0) tool, which evaluates five key domains: (1) the randomization process, (2) deviations from intended interventions, (3) missing outcome data (4), outcome measurement, and (5) selective reporting of results. Each domain was judged as “low risk,” “some concerns,” or “high risk,” and an overall risk-of-bias judgment was subsequently derived for each study (23).

### 2.6 Statistical analysis

A frequentist network meta-analysis was conducted using Stata 17.0. Network plots were constructed to illustrate the geometry of available direct comparisons, followed by network meta-analyses for each outcome. Global inconsistency was first assessed; a consistency model was applied when no significant inconsistency was detected (P > 0.05), whereas an inconsistency model was used when inconsistency was significant (P ≤ 0.05) (24, 25). When closed loops with both direct and indirect evidence were present, local inconsistency was further explored using the node-splitting approach (26). All outcomes were continuous variables and summarized using mean differences (MD) for outcomes measured on the same scale or standardized mean differences (SMD) for outcomes measured on different scales. For multi-arm trials, shared comparator groups were appropriately split to avoid double counting (24). When both baseline and endpoint data were available, change scores were preferentially used; otherwise, endpoint values were analyzed. Standard deviations missing but reported as standard errors, 95% confidence intervals, or interquartile ranges were converted according to the Cochrane Handbook (27). Effect directions were harmonized so that positive SMD values consistently indicated beneficial intervention effects. Psychological outcomes (depression, anxiety, and stress) were interpreted according to their original scoring direction, with lower scores reflecting symptom improvement. For physical function outcomes, measures in which lower values indicated better performance were inverted prior to analysis to ensure directional consistency. Intervention ranking was estimated using the surface under the cumulative ranking curve (SUCRA), with higher values indicating a greater probability of being the most effective intervention (28). Results were presented as forest plots, league tables, and ranking plots, and comparison-adjusted funnel plots were used to assess potential publication bias and small-study effects (25, 29).

### 2.7 Sensitivity analysis

Leave-one-out sensitivity analyses were performed by sequentially excluding each study and recalculating the pooled effect estimate using a random-effects model. The results indicated that the overall effect was largely driven by one influential study, while exclusion of other studies did not materially change the direction of the pooled estimate (30).

### 2.8 Quality of evidence assessment (CINeMA Assessment)

To ensure objectivity and consistency, two reviewers independently conducted the assessments and documented their judgments in the data extraction form. Any discrepancies were resolved through discussion or consultation with a third reviewer. The results were summarized in graphical and tabular formats and served as the basis for subsequent quality of evidence evaluations using the CINeMA frameworks (31, 32).

## 3 Results

### 3.1 Literature search and study selection

**Figure 1:**
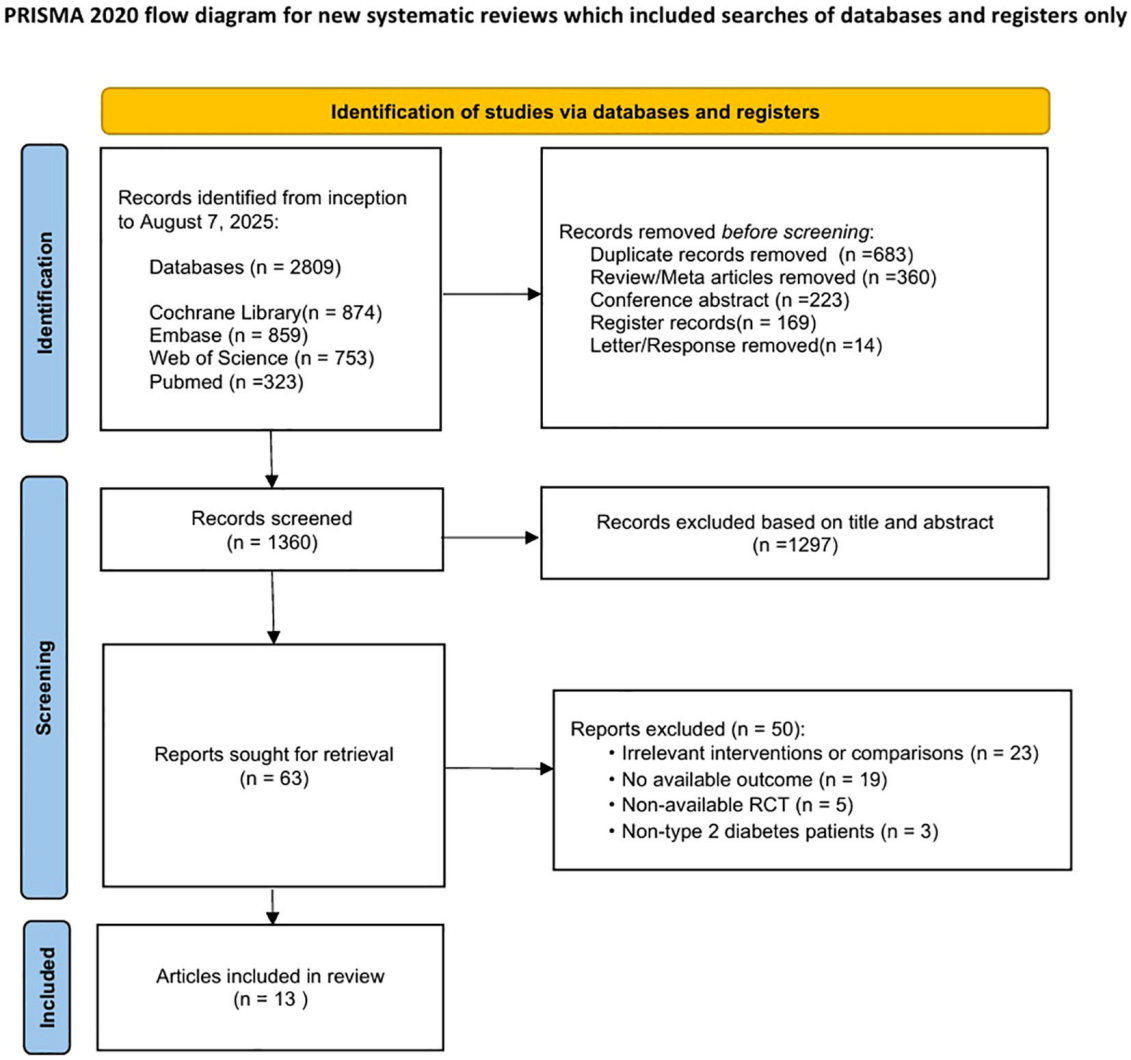
PRISMA 2020 flow diagram of the study selection process.

A total of 2,809 records were identified through database searching (Cochrane Library, n = 874; Embase, n = 859; Web of Science, n = 753; PubMed, n = 323). After removal of duplicate records (n = 683), review and meta-analysis articles (n = 360), conference abstracts (n = 223), trial registry records (n = 169), and letters or correspondence (n = 14), 1,360 records remained for title and abstract screening. Following initial screening, 1,297 records were excluded for not meeting the eligibility criteria, leaving 63 articles for full-text assessment. Of these, 46 studies were further excluded due to irrelevant interventions or comparators (n = 23), absence of extractable outcome data (n = 19), non-randomized study design (n = 5), or inclusion of participants without type 2 diabetes mellitus (n = 3). Ultimately, 13 randomized controlled trials (RCTs) (33–45) were included in the systematic review and network meta-analysis. The study selection process is illustrated in **Figure 1**.

### 3.2 Characteristics of included studies

A total of 13 randomized controlled trials (RCTs), published between 2005 and 2024, were ultimately included, comprising approximately more than 500 participants with type 2 diabetes mellitus (T2DM). The studies were conducted across multiple countries, including the United States, China, Iran, Japan, Thailand, Brazil, Germany, and Australia, providing reasonable international representation. Sample sizes of individual trials ranged from 15 to 120 participants. The age of participants varied widely, spanning from adolescents aged 14–17 years at high risk of T2DM to older adults aged over 78 years, with most studies focusing on middle-aged and older populations. Female participants predominated across studies, and several trials exclusively enrolled women. T2DM diagnosis was primarily based on American Diabetes Association (ADA) criteria or clinical diagnosis by physicians, with some studies additionally applying thresholds for glycated hemoglobin (HbA1c) or fasting plasma glucose. Diabetes duration ranged from newly diagnosed or not explicitly reported to more than 10 years. Baseline HbA1c levels generally indicated suboptimal glycaemic control, ranging approximately from 6.1% to 9.0%. Interventions mainly consisted of distinct mind–body and exercise-related approaches, including yoga (2 trials), mindfulness-based interventions (3 trials), Tai chi (2 trials), Pilates (1 trial), Yi Ren Medical Qigong–based mind–body exercise (YRMQ; 1 trial), Walking Meditation (1 trial), Laughter yoga (1 trial), Biofeedback-Assisted Relaxation Training (BFRT; 1 trial), Progressive Resistance Training (PRT; 1 trial), and Guided Imagery Training (GI; 1 trial). Intervention durations most commonly ranged from 8 to 16 weeks, with the longest lasting up to 6 months. Intervention frequency was typically 1–3 sessions per week, with session durations of 30–90 minutes. Control conditions included usual medical care, diabetes education, lifestyle advice, wait-list control, or sham exercise/low-intensity stretching. Outcome measures covered both physical function and psychological domains. Physical outcomes included walking capacity, balance, aerobic fitness, and muscle strength (e.g., the 6-minute walk test, 10-m walk test, balance assessments, and strength measures), while psychological outcomes primarily focused on depression, anxiety, and perceived stress, commonly assessed using validated instruments such as the BDI/BDI-II, STAI, and PSS. Detailed study characteristics are presented in **Supplementary 2**.

### 3.3 Risk of bias assessment

According to the Risk of Bias 2.0 (RoB 2.0) assessment, most included studies were judged to be at low risk of bias in the domains of the randomization process, missing outcome data, and selection of the reported results. However, some concerns were commonly identified in the domains of bias due to deviations from intended interventions and bias in measurement of outcomes. Overall, the majority of studies were rated as having some concerns in terms of overall risk of bias, with only a small number of trials classified as low risk. The detailed RoB 2.0 assessments are presented in **Supplementary 4A**.

### 3.4 Network meta-analysis results

#### 3.4.1 Overall inconsistency assessment

Before conducting the network meta-analysis, a global inconsistency test was first performed for each outcome. The results showed that no significant inconsistency was observed for balance ability, walking ability, depression, anxiety, or stress outcomes (P > 0.05); therefore, consistency models were applied for these outcomes. In contrast, significant inconsistency was detected for aerobic capacity and muscle strength outcomes (P < 0.01), and inconsistency models were accordingly adopted. Detailed results of the global inconsistency tests are presented in **Supplementary 3**.

#### 3.4.2 Balance ability

For balance ability, a network meta-analysis was conducted using a consistency model. SUCRA rankings indicated that Pilates ranked highest (SUCRA = 100.0%, mean rank = 1.0), followed by the control group (27.7%, mean rank = 2.4) and Tai chi (22.3%, mean rank = 2.6).

Pairwise comparisons showed that Pilates significantly improved balance ability compared with control (SMD = 1.52, 95% CI: 0.77 to 2.28), whereas no significant difference was observed between Tai chi and control (SMD = −0.03, 95% CI: −0.41 to 0.34). In direct comparisons between interventions, Pilates was significantly superior to Tai chi (SMD = −1.55, 95% CI: −2.39 to −0.71), suggesting a potential relative advantage of Pilates in improving balance ability. Detailed results are provided in **Supplementary 5.1**.

#### 3.4.3 Aerobic capacity

For aerobic capacity, analyses were performed using an inconsistency model. SUCRA rankings showed that Walking Meditation ranked highest (SUCRA = 99.9%, mean rank = 1.0), whereas the control group (28.4%) and Tai chi (21.7%) ranked relatively lower.

Pairwise comparisons demonstrated that Walking Meditation significantly improved aerobic capacity compared with control (SMD = 1.40, 95% CI: 0.44 to 2.37), and also showed a more favorable effect compared with Tai chi (SMD = 1.46, 95% CI: 0.59 to 2.33). In contrast, no significant difference was observed between Tai chi and control (SMD = −0.05, 95% CI: −0.76 to 0.65). Detailed results are shown in **Supplementary 5.2**.

#### 3.4.4 Walking ability

For walking ability, a consistency model was used for the network meta-analysis. SUCRA rankings indicated that Pilates ranked highest (SUCRA = 98.2%, mean rank = 1.0), followed by Tai chi (46.4%, mean rank = 2.1), while the control group ranked lowest (5.4%).

Pairwise comparisons showed that Pilates demonstrated a favorable improvement compared with control (SMD = 1.20, 95% CI: 0.28 to 2.12), whereas no statistically significant difference was observed between Tai chi and control (SMD = 0.23, 95% CI: −0.13 to 0.59). In direct comparisons between interventions, the difference between Pilates and Tai chi did not reach statistical significance (SMD = −0.97, 95% CI: −1.96 to 0.02), indicating uncertainty regarding their relative effects on walking ability. Detailed results are presented in **Supplementary 5.3**.

#### 3.4.5 Muscle strength

For muscle strength, analyses were conducted using an inconsistency model. SUCRA rankings indicated that Yoga ranked highest (SUCRA = 92.6%, mean rank = 1.2), followed by Walking Meditation (53.3%, mean rank = 2.4) and Tai chi (41.4%, mean rank = 2.8), while the control group ranked lowest (12.6%).

Pairwise comparisons showed that Yoga demonstrated a favorable improvement compared with control (SMD = 0.79, 95% CI: 0.26 to 1.33), whereas no statistically significant differences were observed between Tai chi and control (SMD = 0.19, 95% CI: −0.18 to 0.56) or between Walking Meditation and control (SMD = 0.34, 95% CI: −0.49 to 1.16). Direct comparisons among different interventions also failed to reach statistical significance, suggesting that the relative effects among interventions remain unclear. Detailed results are provided in **Supplementary 5.4**.

#### 3.4.6 Depression

For depression, a consistency model was applied in the network meta-analysis. SUCRA rankings indicated that PRT ranked relatively higher (SUCRA = 60.5%, mean rank = 2.6), followed by YRMQ (51.2%) and mindfulness-based interventions (51.0%), whereas BFRT and the control group ranked relatively lower.

Pairwise comparisons showed that no statistically significant differences were observed between any intervention and control or BFRT. Moreover, direct comparisons among different interventions showed inconsistent effect directions and wide confidence intervals, indicating substantial uncertainty regarding the intervention effects on depression outcomes. Detailed results are shown in **Supplementary 5.5**.

#### 3.4.7 Anxiety

For anxiety, a consistency model was used for analysis. SUCRA rankings indicated that BFRT ranked relatively high (SUCRA = 90.4%, mean rank = 1.3).

Pairwise comparisons demonstrated that mindfulness-based interventions showed more favorable effect estimates compared with control (SMD = 1.05, 95% CI: 0.46 to 1.63) and compared with BFRT (SMD = 1.34, 95% CI: 0.37 to 2.31), whereas comparisons between yoga and control or BFRT did not reach statistical significance. Direct comparisons suggested that the relative effects between yoga and mindfulness-based interventions remain unclear. Detailed results are presented in **Supplementary 5.6**.

#### 3.4.8 Stress

For stress, a consistency model was applied in the network meta-analysis. SUCRA rankings indicated that Mindfulness-based interventions ranked relatively higher (SUCRA = 72.2%, mean rank = 2.4), followed by YRMQ and PRT, while GI and the control group ranked relatively lower. Pairwise comparisons showed that no statistically significant differences were observed between any intervention and control. In addition, direct comparisons among different interventions showed inconsistent effect directions and wide confidence intervals, indicating that the intervention effects on stress outcomes remain difficult to distinguish. Detailed results are provided in **Supplementary 5.7**.

#### 3.4.9 Certainty of evidence and interpretation

It should be noted that most comparisons in the present study were based on a limited number of randomized controlled trials with small sample sizes, and several outcomes exhibited notable inconsistency or imprecision. CINeMA assessments indicated that the overall certainty of evidence ranged from low to very low. Therefore, the findings of this network meta-analysis should be considered preliminary and exploratory, primarily intended to suggest potential intervention directions rather than to establish definitive comparative effectiveness. Further high-quality, large-scale randomized controlled trials are needed to confirm these findings.

### 3.5 Sensitivity analysis

Leave-one-out sensitivity analyses indicated variable robustness across outcomes. Muscle strength and balance outcomes remained stable after exclusion of any single study, whereas depression and stress outcomes were sensitive to influential trials, particularly study 9 (and study 23 for stress), with effect estimates becoming non-significant after exclusion. Anxiety and walking ability showed consistent directions of effect, although the precision of the estimates was influenced by the removal of individual studies. In contrast, aerobic capacity results were highly unstable due to the inclusion of only two trials. Overall, several outcomes were sensitive to individual studies and should therefore be interpreted with caution. Detailed results are presented in **Supplementary 6**.

### 3.6 Publication bias

Funnel plot inspection suggested that the overall distributions of studies across outcomes were largely symmetrical, indicating an acceptable risk of publication bias. For balance ability and muscle strength outcomes, the distribution of study points showed slight asymmetry, suggesting the possible presence of small-study effects. In contrast, the funnel plots for aerobic capacity and walking ability appeared relatively regular and symmetrical, with no clear evidence of publication bias. Among psychological outcomes, the distribution of studies for depression was relatively scattered with noticeable asymmetry, indicating a potential risk of publication bias, while the funnel plot for anxiety showed mild asymmetry, possibly attributable to limited sample sizes. The funnel plot for stress outcomes demonstrated an overall symmetrical distribution with evenly dispersed study points, suggesting no obvious publication bias. Detailed funnel plots are provided in **Supplementary 5D**.

### 3.7 Quality of evidence assessment (CINeMA Assessment)

Quality of evidence assessment using the CINeMA framework showed marked variability in the credibility of network comparisons across outcomes. Overall, the certainty of evidence was predominantly low to very low, although some direct comparisons reached moderate or high confidence. For balance ability, the comparison between control and Tai chi (CON vs. Tai chi) was rated as high confidence, while that between control and Pilates (CON vs. Pilates) was rated as moderate confidence; the indirect comparison between Pilates and Tai chi was rated as very low confidence due to serious heterogeneity and inconsistency. For aerobic capacity, evidence was mainly derived from single studies, with the control versus Tai chi comparison rated as moderate confidence and most other comparisons rated as low to very low confidence because of imprecision and network inconsistency. For walking ability and muscle strength, most comparisons were rated as low or very low confidence, largely owing to limited study numbers, imprecision, and heterogeneity. For psychological outcomes (depression, anxiety, and stress), overall certainty was low, with direct comparisons mostly rated as low confidence and indirect comparisons almost uniformly rated as very low confidence due to serious inconsistency and imprecision. Detailed assessments are provided in **Supplementary 4B**.

## 4 Discussion

### 4.1 Principal findings

This study compared the relative effects of multiple mind–body exercise interventions on physical function and psychological outcomes using network meta-analysis. The findings indicated that potential advantages varied across outcomes, with Pilates ranking higher for balance and walking ability, Walking Meditation for aerobic capacity, and Yoga for muscle strength, whereas differences among interventions were generally limited for psychological outcomes (depression, anxiety, and stress). Importantly, most comparisons were informed by a small number of randomized controlled trials, and several outcomes were affected by inconsistency or imprecision. Moreover, CINeMA assessments indicated that the overall certainty of evidence ranged from low to very low. Therefore, the present findings should be interpreted primarily as exploratory, intended to highlight potential intervention signals rather than to establish definitive comparative effectiveness.

### 4.2 Comparison with previous studies

Compared with previous studies, the findings of the present analysis show both consistency and incremental contributions across several domains. A 12-week Pilates program has been reported to significantly improve glycaemic control, muscle strength, gait speed, balance, and flexibility in older adults with type 2 diabetes mellitus (T2DM) (46). Building on this evidence, the present network meta-analysis further supports the relative advantage of Pilates in improving balance and walking ability. With regard to mindfulness-based interventions, prior systematic reviews have predominantly focused on populations with general chronic conditions or psychological disorders, including university students, and have consistently demonstrated significant effects in reducing anxiety and stress (47–49). In contrast, our findings indicate that Mindfulness-based interventions produced significantly greater improvements in anxiety among patients with diabetes when compared with both BFRT and control conditions, supporting the potential value of mindfulness approaches in diabetes-related mental health management. For Tai chi, previous literature has reported benefits in enhancing balance and reducing fall risk (50). However, in the present analysis, Tai Chi demonstrated effects on balance ability that were comparable to those of conventional care, with no statistically significant differences observed. Regarding YRMQ, Laughter yoga, and BFRT, existing evidence is largely derived from small, single trials with inconsistent findings. Although our results suggested potential improvement trends for these interventions, the overall quality of evidence remained low, highlighting the need for further large-scale, high-quality randomized controlled trials to clarify their effectiveness.

### 4.3 Potential mechanisms

The present findings suggest that mind–body exercise may confer dual benefits for patients with type 2 diabetes mellitus (T2DM) through a coordinated neuro–muscular–immune–metabolic axis. At a mechanistic level, these benefits appear to arise from the simultaneous modulation of skeletal muscle metabolism and central neural–emotional regulatory circuits. On the one hand, regular low- to moderate-intensity exercise modalities such as Pilates and Tai chi can activate the PGC-1α/AMPK signaling pathway (51), thereby improving mitochondrial function and glucose utilization efficiency, These adaptations may subsequently enhance muscle strength and overall physical performance (52). On the other hand, interventions represented by mindfulness training and yoga may attenuate hyperactivation of the hypothalamic–pituitary–adrenal (HPA) axis and sympathetic nervous system (53), leading to reduced cortisol secretion and enhanced inhibitory control of limbic regions by the prefrontal cortex. These neural adaptations contribute to alleviation of anxiety and depressive symptoms, while concurrently downregulating pro-inflammatory cytokines such as IL-6 and TNF-α and upregulating anti-inflammatory mediators including IL-10 and regulatory T-cell (Treg) activity. Together, these processes promote neuroplasticity and suppress chronic low-grade inflammation (54).

Differential effects across interventions may reflect their distinct physiological emphases. Pilates places greater emphasis on core stability and proprioceptive feedback (10), which may explain its superior performance in improving balance and walking ability. Walking Meditation ranked relatively higher for aerobic capacity, likely because sustained walking-based aerobic activity activates muscle energy homeostasis pathways (e.g., AMPK/PGC-1α signaling), enhances mitochondrial biogenesis, and improves oxidative metabolic efficiency, thereby facilitating cardiorespiratory endurance adaptations (55). Yoga may show greater benefits for muscle strength, as the isometric contractions and anti-gravity postures commonly incorporated in yoga practice improve neuromuscular control and muscle fiber coordination, while simultaneously promoting exercise-induced muscular adaptations that enhance strength output (56). Mindfulness-based interventions primarily alleviate anxiety through cognitive–emotional regulation mechanisms (57). Overall, these interventions appear to disrupt the vicious cycle of stress, metabolic dysregulation, and functional impairment through multiple interacting pathways, thereby supporting integrated improvements in both psychological and physical function.

### 4.4 Clinical implications

Despite the overall low certainty of evidence, the present findings provide exploratory clinical insights for the management of patients with type 2 diabetes mellitus (T2DM). Different mind–body exercise interventions showed outcome-specific potential advantages across physical function domains, suggesting that exercise prescriptions may be tailored to patients’ predominant functional limitations rather than relying on a single uniform modality. Interventions emphasizing postural control and core stability may be more suitable for individuals with functional decline or elevated fall risk, whereas rhythmic or sustained activities may better support improvements in cardiorespiratory endurance.

For psychological outcomes, between-intervention differences were generally modest and uncertain; however, relatively favorable effects observed for anxiety- and stress-related outcomes suggest that integrating psychological regulation components into exercise programs may help address the mental health burden commonly experienced by patients with T2DM. Overall, these findings underscore the potential value of individualized, multidisciplinary, mind–body–integrated approaches, while emphasizing that the results should be interpreted as supportive, exploratory evidence rather than definitive recommendations.

### 4.5 Limitations and future directions

Several limitations should be acknowledged. Only 13 RCTs with fewer than 800 participants were included, limiting statistical power and precluding meta-regression or subgroup analyses to explore potential effect modifiers (e.g., age, sex, intervention type, or disease duration). In addition, the included trials encompassed a wide age range and heterogeneous clinical profiles, spanning from adolescents at high risk of type 2 diabetes mellitus to older adults with long-standing disease and diabetes-related complications. Such demographic and clinical heterogeneity may limit the generalizability of the findings and could partly explain the inconsistency or imprecision observed across certain outcomes. Methodological quality was generally modest, with limited blinding and substantial heterogeneity in intervention frequency, duration, intensity, and adherence reporting. Psychological outcomes were assessed using diverse instruments, reducing cross-study comparability, and most interventions were short term (8–24 weeks), with no long-term follow-up to assess sustained effects.

Future studies should focus on large, multicenter, high-quality RCTs with improved methodological rigor, standardized intervention parameters and outcome measures, and longer follow-up periods. Mechanistic investigations and adequately powered subgroup analyses are also needed to clarify differential benefits across populations and to support more precise, individualized intervention strategies.

## 5 Conclusion

Using a network meta-analysis, this study systematically compared the relative effects of multiple mind–body interventions on physical function and psychological outcomes in patients with type 2 diabetes mellitus. The results indicated that potential advantages varied by outcome, with Pilates ranking higher for balance and walking ability, Walking Meditation for aerobic capacity, and Yoga for muscle strength, whereas differences among interventions were generally limited and unstable for psychological outcomes, including depression, anxiety, and stress. Given the limited number of included studies, suboptimal methodological quality, and substantial heterogeneity in interventions and outcome measures, the overall certainty of the evidence was low, and the findings should be interpreted as exploratory. Nevertheless, this study provides a structured synthesis of existing evidence on mind–body interventions in the comprehensive management of type 2 diabetes and offers a reference framework for future high-quality research and informed clinical decision-making.

## Data Availability

The original contributions presented in the study are included in the article/**Supplementary Material**. Further inquiries can be directed to the corresponding author.
