# Supplementary material for: Mind–body exercise for improving balance, aerobic capacity, walking ability, muscle strength, and mental health in patients with type 2 diabetes: a network meta-analysis
Source: Front Endocrinol (Lausanne). 2026 Mar 4;17:1749651. doi: 10.3389/fendo.2026.1749651 (PMC12995793; doi:10.3389/fendo.2026.1749651)
Supplement: Supplementary file 2 [file Table2.docx]

Supplementary Material

# Search Strategies Per Database

**A:PubMed**

| **Database: PubMed** **(323)** | |
| --- | --- |
| #1 | "Mind-Body Therapies"[Mesh] |
| #2 | ((((((((((((mind-body practice[Title/Abstract]) OR (yoga[Title/Abstract])) OR (tai chi[Title/Abstract])) OR (baduanjin[Title/Abstract])) OR (liuzijue[Title/Abstract])) OR (wuqinxi[Title/Abstract])) OR (qigong[Title/Abstract])) OR (moving meditation[Title/Abstract])) OR (pilates[Title/Abstract])) OR (barre workout[Title/Abstract])) OR (dance therapy[Title/Abstract])) OR (aqua yoga[Title/Abstract])) OR (mindfulness training[Title/Abstract]) |
| #3 | "Diabetes Mellitus"[Mesh] |
| #4 | (((((((((diabetes[Title/Abstract]) OR (diabetes mellitus, experimental[Title/Abstract])) OR (type 1 diabetes mellitus[Title/Abstract])) OR (wolfram syndrome[Title/Abstract])) OR (type 2 diabetes mellitus[Title/Abstract])) OR (lipoatrophic diabetes mellitus[Title/Abstract])) OR (gestational diabetes[Title/Abstract])) OR (donohue syndrome[Title/Abstract])) OR (latent autoimmune diabetes in adults[Title/Abstract])) OR (prediabetic state[Title/Abstract]) |
| #5 | "Randomized Controlled Trial" [Publication Type] |
| #6 | ((((((controlled clinical trial[Title/Abstract]) OR (controlled trial[Title/Abstract])) OR (controlled study[Title/Abstract])) OR (controlled clinical study[Title/Abstract])) OR (RCT[Title/Abstract])) OR (randomiz*[Title/Abstract])) OR (randomis*[Title/Abstract]) |
| #7 | #1 OR #2 |
| #8 | #3 OR #4 |
| #9 | #5 OR #6 |
| #10 | #7 AND #8 AND #9 |

**B:Web of Science**

| **Database: Web of Science (753)** | |
| --- | --- |
| #1 | Ts=（Mind-Body Therapies OR Mind-Body Exercise OR Mind-Body Practice OR Yoga OR Tai Chi OR Baduanjin OR Liuzijue OR Wuqinxi OR Qigong OR Moving Meditation OR Pilates OR Barre Workout OR Dance Therapy OR Aqua Yoga OR Mindfulness Training） |
| #2 | Ts =（Diabetes Mellitus OR Diabetes OR Diabetes Mellitus, Experimental OR Diabetes Mellitus, Type 1 OR Wolfram Syndrome OR Diabetes Mellitus, Type 2 OR Diabetes Mellitus, Lipoatrophic OR Diabetes, Gestational OR Donohue Syndrome OR Latent Autoimmune Diabetes in Adults OR Prediabetic State） |
| #3 | Ts =（randomized controlled trial or controlled clinical trial or controlled trial or controlled study or controlled clinical study or RCT or randomiz* or randomis* or placebo or randomly or trial or groups） |
| #4 | #3 AND #2 AND #1 |

**C:Embase**

| **Database: Embase** **(859)** | |
| --- | --- |
| #1 | 'alternative medicine'/exp |
| #2 | 'mind-body exercise':ti,ab OR 'mind-body practice':ti,ab OR 'yoga':ti,ab OR 'tai chi':ti,ab OR 'baduanjin':ti,ab OR 'liuzijue':ti,ab OR 'wuqinxi':ti,ab OR 'qigong':ti,ab OR 'moving meditation':ti,ab OR 'pilates':ti,ab OR 'barre workout':ti,ab OR 'dance therapy':ti,ab OR 'aqua yoga':ti,ab OR 'mindfulness training':ti,ab |
| #3 | 'diabetes mellitus'/exp |
| #4 | 'diabetes mellitus':ti,ab OR 'diabetes':ti,ab OR 'diabetes mellitus, experimental':ti,ab OR 'type 1 diabetes mellitus':ti,ab OR 'wolfram syndrome':ti,ab OR 'type 2 diabetes mellitus':ti,ab OR 'lipoatrophic diabetes mellitus':ti,ab OR 'gestational diabetes':ti,ab OR 'donohue syndrome':ti,ab OR 'latent autoimmune diabetes in adults':ti,ab OR 'prediabetic state':ti,ab |
| #5 | 'randomized controlled trial'/exp |
| #6 | 'controlled clinical trial':ab,ti OR 'controlled trial':ab,ti OR 'controlled study':ab,ti OR 'controlled clinical study':ab,ti OR rct:ab,ti OR randomiz*:ab,ti OR randomis*:ab,ti |
| #7 | #1 OR #2 |
| #8 | #3 OR #4 |
| #9 | #5 OR #6 |
| #10 | #7 AND #8 AND #9 |

**D:Cochrane Library**

| **Database: Cochrane Library** **(859)** | |
| --- | --- |
| #1 | MeSH descriptor: [Complementary Therapies] explode all trees |
| #2 | "mind-body exercise" OR"mind-body practice" OR"yoga" OR"tai chi" OR"baduanjin" OR"liuzijue" OR"wuqinxi" OR"qigong" OR"moving meditation" OR"pilates" OR"barre workout" OR"dance therapy" OR"aqua yoga" OR"mindfulness training" |
| #3 | MeSH descriptor: [Diabetes Mellitus] explode all trees |
| #4 | "diabetes mellitus" OR"diabetes" OR"diabetes mellitus, experimental" OR"type 1 diabetes mellitus" OR"wolfram syndrome" OR"type 2 diabetes mellitus" OR"lipoatrophic diabetes mellitus" OR"gestational diabetes" OR"donohue syndrome" OR"latent autoimmune diabetes in adults" OR"prediabetic state" |
| #5 | MeSH descriptor: [Randomized Controlled Trial] explode all trees |
| #6 | 'controlled clinical trial':ab,ti OR 'controlled trial':ab,ti OR 'controlled study':ab,ti OR 'controlled clinical study':ab,ti OR rct:ab,ti OR randomiz*:ab,ti OR randomis*:ab,ti |
| #7 | #1 OR #2 |
| #8 | #3 OR #4 |
| #9 | #5 OR #6 |
| #10 | #7 AND #8 AND #9 |

# Basic information table of literature

| No. | Study  (Country) | RCT | Expl  (n,) | Con  (n) | Sex | Age | T2DM diagnostic criteria | Duration of T2DM | Baseline HbA1c | Exp Cat. | Exp Details | Con Cat. | Con Details | Outcomes |
| --- | --- | --- | --- | --- | --- | --- | --- | --- | --- | --- | --- | --- | --- | --- |
| 4 | Karla Cinara Bezerra Melo 2020(Brazil) | Yes | 11 | 11 | All female | Intervention: 65.5 ± 5.2 y; Control: 67.5 ± 6.3 y | Clinically diagnosed T2DM (ADA criteria) | Intervention: 9.1 ± 6.1 y;  Control: 8.3 ± 4.4 y | Pilates: 7.8 ± 1.0 %;  Control: 7.4 ± 0.5 % | Pilates | Pilates: 3 sessions/week, 60 min/session, 12 weeks; moderate intensity (RPE 6–12/15); dynamic and isometric exercises plus stretching; progressed every 3 weeks,3 times/week,12 weeks | CON | No exercise intervention; usual treatment and diet control. | Walking ability (10-Meter Walk Test) Balance (Sit-to-Stand, Rising from Chair and Walking) |
| 5 | B.C. Bock2020(USA) | Yes | 24 | 23 | Yoga: 62.5% female (n=15); SE: 62.5% female | Yoga: 57.5 ± 6.7 y; SE: 53.8 ± 10.3 | Laboratory-confirmed HbA1c ≥ 6.5% | ≥6 months (inclusion); actual duration not reported | Not reported | Yoga | Iyengar yoga: 2 sessions/week, 60 min/session, 12 weeks; including asanas, static/dynamic practice, breath control, meditation, relaxation; props and DVD for home practice.,2 times/week,12 weeks | CON | Standard exercise (SE): 2 sessions/week, 60 min; walking, cycling, elliptical, aerobics; 5-min warm-up, 50-min moderate intensity (Borg 11–13), 5-min cool-down; supervised by instructor. | Anxiety (Diabetes-39, Anxiety & Worry subscale) |
| 9 | Shu-Ming Chen 2021(China) | Yes | 60 | 60 | Exp: 22 M (35.4%), 40 F (64.6%); Con: 23 M (34.8%), 43 F (65.2%) | Exp: 78.85 ± 7.62 y; Con: 78.95 ± 7.12 y | Diagnosed T2DM (ADA/WHO/ICD criteria not specified) | Not specified; diagnosed T2DM, institutionalized ≤12 months | Intervention: 8.0 ± 1.8 %; Control: 6.6 ± 1.9 % | Mindfulness | Mindfulness program: 9 weeks, 9 sessions, 90 min each; meditation, breathing, body scan, stress management, emotional regulation; delivered by trained nurses in long-term care facility,1 times/week,9 weeks | CON | Usual care: standard diabetes management, regular check-ups, dietary advice; no mindfulness content. | Depression (Depression Anxiety Stress Scale-21) Stress (Relocation Stress Scale) |
| 13 | Mohammad Ali Faridi Dastjerdi 2024(Iran) | Yes | 15 | 15 | Not specified (adults aged 41–69) | Exp: 57.5 ± 9.2 y; Con: 54.0 ± 5.5 y | Clinically diagnosed T2DM with diabetic neuropathy; FBG > 120 mg/dL | Exp: 9.6 ± 6.6 y; Con: 8.9 ± 7.5 y | Not reported | Yoga | Yoga: 3 sessions/week, 60 min/session, 8 weeks; included Surya Namaskar, Pavanamuktasana, Bhujangasana, Shalabhasana, Ardha Matsyendrasana, pranayama.,3 times/week,8 weeks | CON | Usual daily activities; no yoga. | Muscle strength (Flexor & Extensor strength) |
| 16 | Atikarn Gainey 2016(Thailand) | Yes | 12 | 11 | Exp: 2M/10F; Con: 2M/9F | Exp: 58 ± 3 y; Con: 63 ± 2 y | Clinically diagnosed T2DM; HbA1c 7–9% | Exp: ≥6 y (5/12); Con: ≥6 y (4/11) | 60.0 ± 4.9 vs 60.9 ± 4.0 mmol/mol (SEM) | WalkingMeditation | Walking meditation: treadmill walking with mindfulness cues (“Budd/Dha”), 30 min/session,3 times/week,12 weeks | CON | Traditional treadmill walking; dose-matched, no mindfulness. | Muscle strength (Isometric Back/Leg Dynamometer, Takei Sci) Aerobic capacity (VO₂max) |
| 23 | Mayumi Hirosaki 2023(Japan) | Yes | 21 | 21 | Exp: 14 F (66.7%); Con: 14 F (66.7%) | Exp: 71.8 ± 6.4 y; Con: 70.6 ± 8.2 y | Clinically diagnosed T2DM; HbA1c 6.1%–7.9% | Not reported | 7.07 ± 0.7 %; 7.19 ± 0.7 % | LaughterYoga | Laughter yoga: ~30-min lecture + 60-min yoga; weekly for 4 weeks, then biweekly for 8 weeks  ,Avg. 0.7–1 times/week,12 weeks | CON | Standard medical treatment and lifestyle counseling; no additional intervention. | Stress (Subjective Stress) |
| 32 | Kopf, S. 2014(Germany) | Yes | 53 | 57 | Exp: 40 F, 13 M; Con: 46 F, 11 M | Exp: 58.7 y [56.7–60.8]; Con: 59.3 y [57.2–61.3] | Clinically diagnosed T2DM with early diabetic nephropathy; guideline-based criteria | Not reported | 7.27% [6.99–7.55]; 7.26% [6.96–7.56] | Mindfulness | MBSR: mindfulness meditation, breathing awareness, body scan, yoga, stress management, group discussion; based on Kabat-Zinn program; ~2 h weekly + one full-day retreat, 8 weeks. | CON | Usual care: standard diabetes management; no MBSR. | Stress (PHQ-Stress Scale) |
| 33 | Paul Lam 2008(Australia) | Yes | 28 | 25 | Exp: 13 M, 15 F (46% F); Con: 16 F, 9 M (64% F) | Exp: 63.2 ± 8.6 y; Con: 60.7 ± 12.2 y | Diagnosed T2DM ≥ 6 months; HbA1c > 7% | >10 years: Exp 13 (46.4%), Con 9 (36%) | 9.0 ± 1.4 %; 8.5 ± 1.2 % | TaiChi | Sun-style 20-form Tai Chi, led by certified instructor; 2 sessions/week, 60 min/session, 6 months. | CON | Waiting list; no exercise. | Walking ability (6-Meter Walk Test) |
| 39 | Ronald A. McGinnis 2005(USA) | Yes | 16 | 14 | Exp: 7 M, 9 F; Con: 7 M, 7 F | Mean 52.63 ± 7.6 y | Physician-confirmed T2DM | Not reported | 7.4 ± 1.4 %; 7.0 ± 1.4 % | BFRT | BFRT: biofeedback-assisted relaxation training, 10 sessions (weekly), 45–60 min; EMG/thermal feedback, breathing, imagery, PMR; booster sessions included.1 times/week,12 weeks | CON | Diabetes education: 3 sessions over 3–4 weeks, 60–75 min each; covering diabetes management, diet, exercise, glucose monitoring, complication prevention. | Depression (BDI-II) Anxiety  (STAI) |
| 42 | Carla K. Miller 2014(USA) | Yes | 27 | 25 | Exp: 63% female; Con: 64% female | Exp: 53.9 ± 8.2 y; Con: 54.0 ± 7.0 y | Physician-diagnosed T2DM ≥ 1 year; HbA1c ≥ 7.0% | Exp: 6.9 ± 3.9 y; Con: 5.9 ± 3.4 y | Not reported | Mindfulness | MB-EAT-D: mindfulness-based eating awareness training; 8×90-min group sessions + 2×150-min intensives + 2 follow-ups; duration 3 months. | CON | Smart Choices (SC): DSME-based diabetes self-management education; format and contact time matched to intervention. | Depression (BDI-II) Anxiety  (BAI) |
| 47 | Amy L. Putiri 2012(USA) | Yes | 7 | 8 | 13 M, 19 F (total sample); not divided by group | Mean 56.3 ± 8.1 y | Physician-diagnosed T2DM; HbA1c > 7.5%; FPG > 7.0 mmol/L | Not reported | 7.9 ± 0.8 %; 8.6 ± 1.2 %; 8.8 ± 1.1 % | YRMQ | Exp 1: YRMQ Qigong, 1×/week 60-min group class + ≥2×/week 30-min home practice, 12 weeks. | CON | Standard diabetes care/education; no additional intervention. | Stress (Perceived Stress Scale, PSS) Depression (BDI) |
|  |  |  | 5 |  |  |  |  |  |  | PRT | Exp 2: PRT, progressive resistance training, 1×/week, 60 min, 12 weeks. |  |  |  |
| 56 | Tracey Tsang 2007(Australia) | Yes | 18 | 20 | Overall 78.9% female (Exp: 44%, Con: 70%) | Mean 65 ± 8 y | Physician-confirmed stable T2DM | Mean 65 ± 8 y | 7.1 ± 0.9 %; 6.9 ± 0.9 % | TaiChi | Tai Chi for Diabetes: blended Sun & Yang style; ~45 min/session (10 warm-up + 30 Tai Chi + 5 cool-down); 2 sessions/week, 16 weeks (32 sessions). | CON | Sham exercise (calisthenics and light stretching), no significant physiological benefit; time/frequency matched. | Balance (Chatex Balance System, Balance Index) Aerobic capacity (Six-Minute Walk Test) Muscle strength (Knee extensor strength, Peak Muscle Power, Endurance repetitions) Mobility (Habitual & Maximal Walking Speed) |
| 60 | Marc J Weigensberg 2009(USA) | Yes | 15 | 14 | Approx. 1:1 (DS: 7 M/7 F; GI: 7 M/8 F) | 14–17 y (mean: 16.1 ± 0.95 vs 15.5 ± 1.0) | Clinically diagnosed T2DM (Chinese guidelines) | Adolescents, T2DM risk (exact duration NR) | 7.6 ± 1.1 %; 7.5 ± 1.0 % | GI | Lifestyle education + Interactive Guided Imagery (IGI): 45 min/session (15 min foresight + 20 min imagery + 10–15 min debriefing); included stress relief, exercise, diet, and self-image imagery.1 times/week,12 weeks | CON | Lifestyle education + Digital Storytelling: 45 min/session; individual/group activity, unrelated to health. | Stress (Perceived Stress Scale, PSS-60) |

Intervention abbreviations are as follows: CON, control group; PRT, Progressive Relaxation Training; YRMQ, Yi Ren Medical Qigong; BFRT, Biofeedback-Assisted Relaxation Training; GI, Guided Imagery

# Tests for inconsistency and model selection

| Outcome | Test equation | χ² (df=1) | P-value | Model used |
| --- | --- | --- | --- | --- |
| Balance ability | [_y_C]_cons = 0 | 0.03 | 0.8709 | Consistency model |
| Aerobic capacity | [_y_C]_cons = 0 | 8.09 | 0.0044** | Inconsistency model |
| Walking ability | [_y_C]_cons = 0 | 1.60 | 0.2062 | Consistency model |
| Muscle strength | [_y_D]_cons = 0 | 8.51 | 0.0035 ** | Inconsistency model |
| Depression | - [_y_B]_cons + [_y_E]_cons = 0 | 0.04 | 0.8322 | Consistency model |
| Anxiety | - [_y_B]_cons + [_y_D]_cons = 0 | 2.52 | 0.1123 | Consistency model |
| Stress | [_y_F]_cons = 0 | 0.10 | 0.7496 | Consistency model |

Note: χ² tests were conducted with 1 degree of freedom. P < 0.05 indicates significant inconsistency.

A: Balance ability


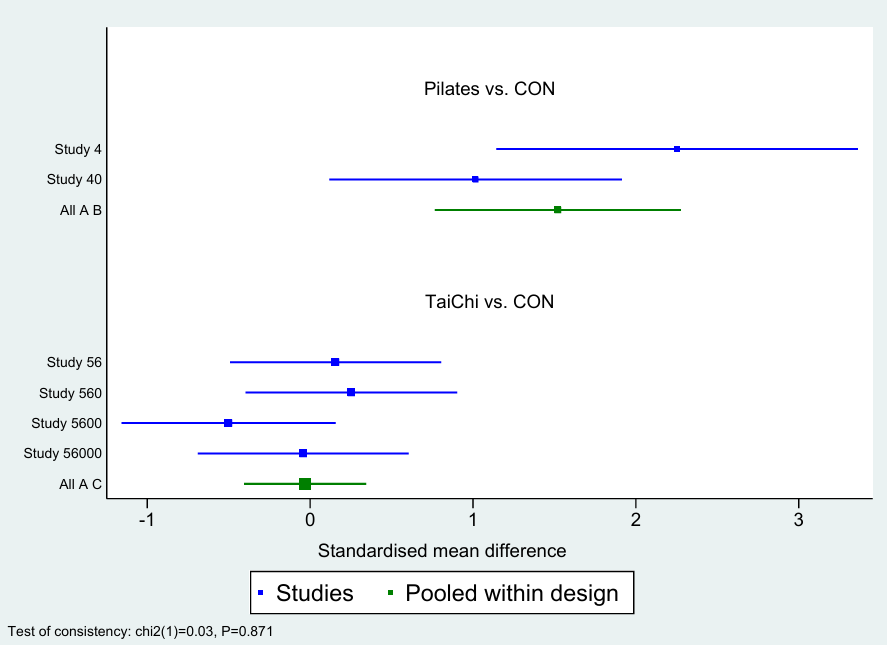


B: Aerobic capacity


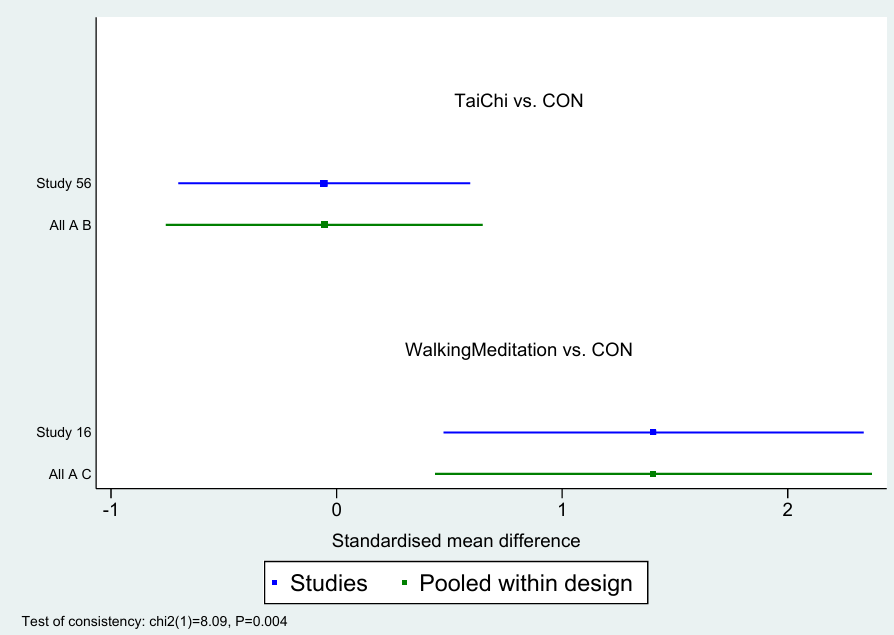


C: Walking ability


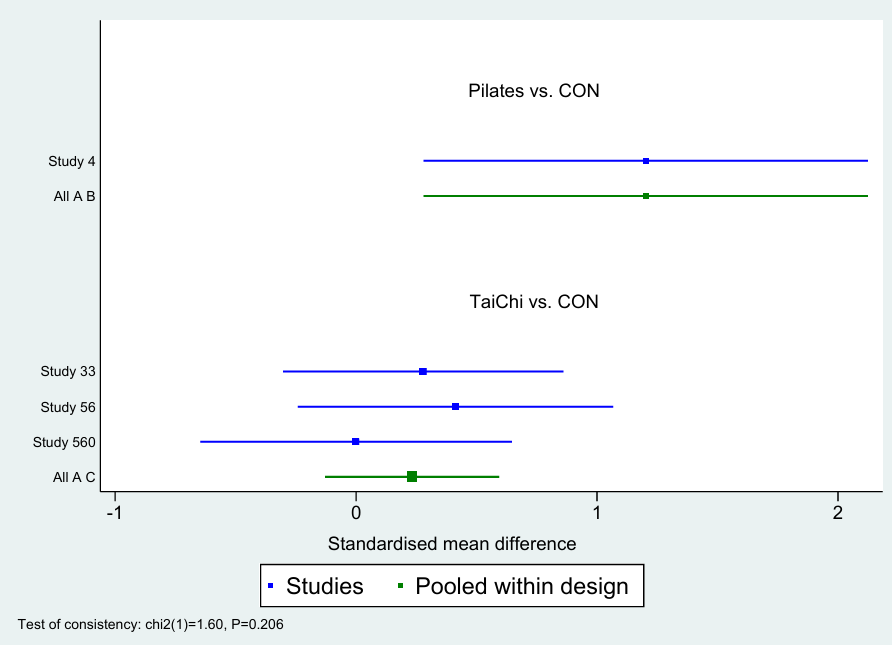


D: Muscle strength


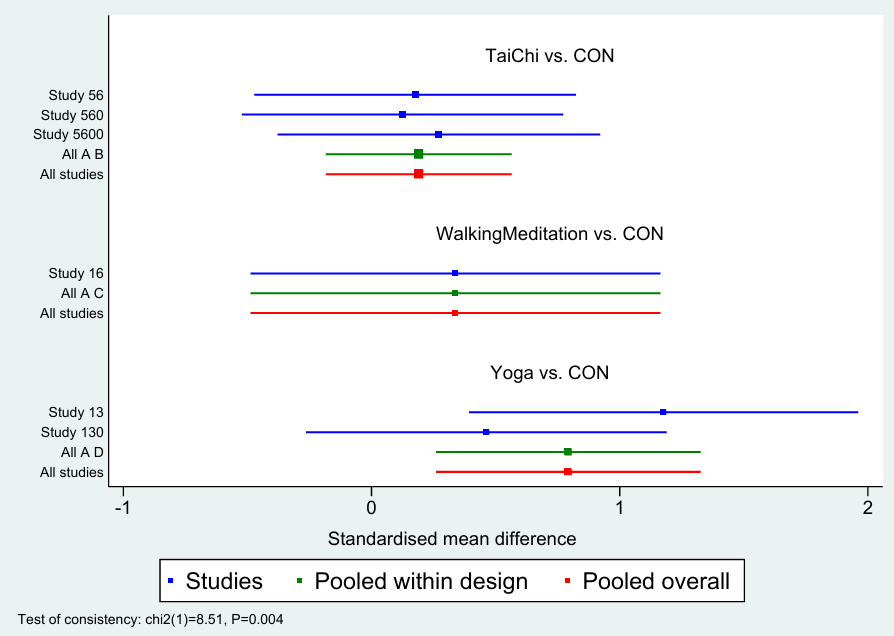


E: Depression


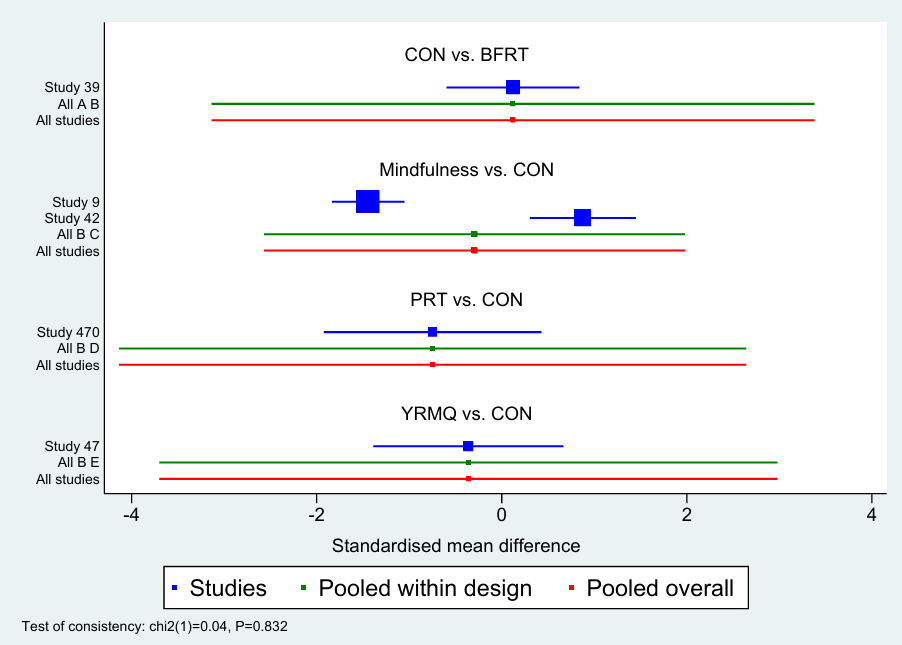


F: Anxiety


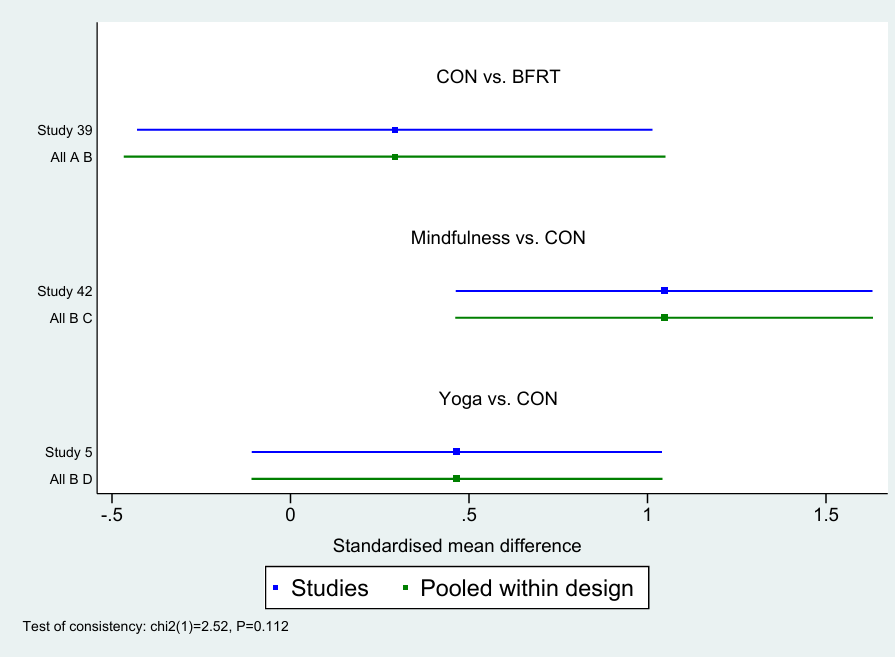


G: Stress


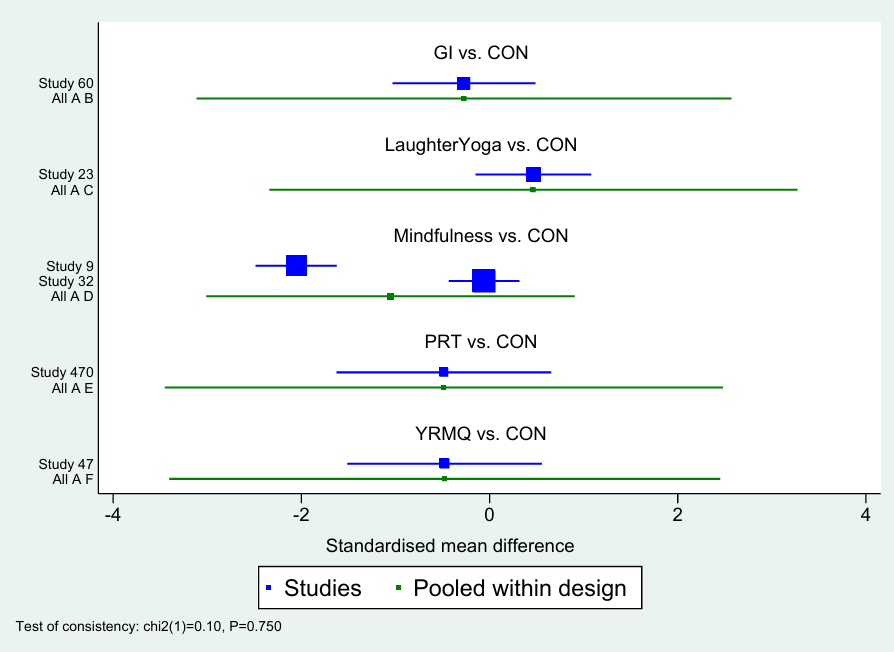


# Risk of bias and CINeMA assessment

A: Risk of bias assessment

| study | Randomization process | Deviations from intended interventions | Mising outcome data | Measurement of the outcome | Selection of the reported result | Overall |
| --- | --- | --- | --- | --- | --- | --- |
| 4 | Low risk | Some concerns | Low risk | Some concerns | Low risk | Some concerns |
| 5 | Low risk | Some concerns | Low risk | Some concerns | Low risk | Some concerns |
| 9 | Some concerns | Some concerns | Low risk | Some concerns | Low risk | Some concerns |
| 13 | Low risk | Some concerns | Low risk | Some concerns | Low risk | Some concerns |
| 16 | Low risk | Some concerns | Some concerns | Some concerns | Low risk | Some concerns |
| 23 | Low risk | Some concerns | Low risk | Low risk | Low risk | Some concerns |
| 31 | Some concerns | Some concerns | Low risk | Some concerns | Low risk | Some concerns |
| 33 | Low risk | Some concerns | Low risk | Low risk | Low risk | Some concerns |
| 39 | Low risk | Some concerns | Low risk | Low risk | Low risk | Some concerns |
| 42 | Low risk | Some concerns | Low risk | Low risk | Low risk | Some concerns |
| 47 | Low risk | Some concerns | Some concerns | Some concerns | Low risk | Some concerns |
| 56 | Low risk | Low risk | Low risk | Low risk | Low risk | Low risk |
| 60 | Low risk | Some concerns | Low risk | Some concerns | Low risk | Some concerns |

B: GRADE Assessment

| Comparison | Number of studies | Within-study bias | Reporting bias | Indirectness | Imprecision | Heterogeneity | Incoherence | Confidence rating |
| --- | --- | --- | --- | --- | --- | --- | --- | --- |
| Balance ability |  |  |  |  |  |  |  |  |
| CON:Pilates | 2 | Some concerns | Low risk | No concerns | No concerns | Some concerns | No concerns | Moderate |
| CON:TaiChi | 4 | No concerns | Low risk | No concerns | No concerns | Some concerns | No concerns | High |
| Pilates:TaiChi | 0 | Some concerns | Low risk | No concerns | No concerns | Major concerns | Major concerns | Very low |
| Aerobic capacity |  |  |  |  |  |  |  |  |
| CON:WalkingMeditation | 1 | Some concerns | Low risk | No concerns | Some concerns | No concerns | No concerns | Very low |
| CON:TaiChi | 1 | No concerns | Low risk | No concerns | Some concerns | No concerns | No concerns | Moderate |
| WalkingMeditation: TaiChi | 0 | Some concerns | Low risk | No concerns | Major concerns | Major concerns | Major concerns | Very low |
| Walking ability |  |  |  |  |  |  |  |  |
| CON:Pilates | 1 | Some concerns | Low risk | No concerns | Some concerns | No concerns | No concerns | Low |
| CON:TaiChi | 3 | Some concerns | Low risk | No concerns | Some concerns | Some concerns | No concerns | Very low |
| Pilates:TaiChi | 0 | Some concerns | Low risk | No concerns | Some concerns | Major concerns | Major concerns | Very low |
| Muscle strength |  |  |  |  |  |  |  |  |
| CON : Yoga | 2 | Some concerns | Low risk | No concerns | Some concerns | Some concerns | No concerns | Very low |
| CON : Walking Meditation | 1 | Some concerns | Low risk | No concerns | Some concerns | No concerns | No concerns | Low |
| CON : TaiChi | 3 | Some concerns | Low risk | No concerns | Some concerns | Some concerns | No concerns | Very low |
| Yoga : Walking Meditation | 0 | Some concerns | Low risk | No concerns | Some concerns | Major concerns | Major concerns | Very low |
| Yoga : TaiChi | 0 | Some concerns | Low risk | No concerns | Some concerns | Major concerns | Major concerns | Very low |
| Walking Meditation : TaiChi | 0 | Some concerns | Low risk | No concerns | Some concerns | Major concerns | Major concerns | Low |
| Depression |  |  |  |  |  |  |  |  |
| CON : Mindfulness | 2 | Some concerns | Low risk | No concerns | Some concerns | Some concerns | No concerns | Very low |
| CON : BFRT | 1 | Some concerns | Low risk | No concerns | Some concerns | No concerns | No concerns | Low |
| CON : YRMQ | 1 | Some concerns | Low risk | No concerns | Some concerns | No concerns | No concerns | Low |
| CON : PRT | 1 | Some concerns | Low risk | No concerns | Some concerns | No concerns | No concerns | Low |
| Mindfulness : BFRT | 0 | Some concerns | Low risk | No concerns | Some concerns | Major concerns | Major concerns | Very low |
| Mindfulness : YRMQ | 0 | Some concerns | Low risk | No concerns | Some concerns | Major concerns | Major concerns | Very low |
| Mindfulness : PRT | 0 | Some concerns | Low risk | No concerns | Some concerns | Major concerns | Major concerns | Very low |
| BFRT : YRMQ | 0 | Some concerns | Low risk | No concerns | Some concerns | Major concerns | Major concerns | Very low |
| BFRT : PRT | 0 | Some concerns | Low risk | No concerns | Some concerns | Major concerns | Major concerns | Very low |
| YRMQ : PRT | 0 | Some concerns | Low risk | No concerns | Some concerns | Major concerns | Major concerns | Very low |
| Anxiety |  |  |  |  |  |  |  |  |
| CON : Yoga | 1 | Some concerns | Low risk | No concerns | Some concerns | No concerns | No concerns | Low |
| CON : BFRT | 1 | Some concerns | Low risk | No concerns | Some concerns | No concerns | No concerns | Low |
| CON : Mindfulness | 1 | Some concerns | Low risk | No concerns | Some concerns | No concerns | No concerns | Low |
| Yoga : BFRT | 0 | Some concerns | Low risk | No concerns | Some concerns | Major concerns | Major concerns | Very low |
| Yoga : Mindfulness | 0 | Some concerns | Low risk | No concerns | Some concerns | Major concerns | Major concerns | Very low |
| BFRT : Mindfulness | 0 | Some concerns | Low risk | No concerns | Some concerns | Major concerns | Major concerns | Very low |
| Stress |  |  |  |  |  |  |  |  |
| CON : Mindfulness | 2 | Some concerns | Low risk | No concerns | Some concerns | Some concerns | No concerns | Very low |
| CON : Laughter Yoga | 1 | Some concerns | Low risk | No concerns | Some concerns | No concerns | No concerns | Low |
| CON : YRMQ | 1 | Some concerns | Low risk | No concerns | Some concerns | No concerns | No concerns | Low |
| CON : PRT | 1 | Some concerns | Low risk | No concerns | Some concerns | No concerns | No concerns | Low |
| CON : GI | 1 | Some concerns | Low risk | No concerns | Some concerns | No concerns | No concerns | Low |
| Mindfulness : Laughter Yoga | 0 | Some concerns | Low risk | No concerns | Some concerns | Major concerns | Major concerns | Very low |
| Mindfulness : YRMQ | 0 | Some concerns | Low risk | No concerns | Some concerns | Major concerns | Major concerns | Very low |
| Mindfulness : PRT | 0 | Some concerns | Low risk | No concerns | Some concerns | Major concerns | Major concerns | Very low |
| Mindfulness : GI | 0 | Some concerns | Low risk | No concerns | Some concerns | Major concerns | Major concerns | Very low |
| Laughter Yoga : YRMQ | 0 | Some concerns | Low risk | No concerns | Some concerns | Major concerns | Major concerns | Very low |
| Laughter Yoga : PRT | 0 | Some concerns | Low risk | No concerns | Some concerns | Major concerns | Major concerns | Very low |
| Laughter Yoga : GI | 0 | Some concerns | Low risk | No concerns | Some concerns | Major concerns | Major concerns | Very low |
| YRMQ : PRT | 0 | Some concerns | Low risk | No concerns | Some concerns | Major concerns | Major concerns | Very low |
| YRMQ : GI | 0 | Some concerns | Low risk | No concerns | Some concerns | Major concerns | Major concerns | Very low |
| PRT : GI | 0 | Some concerns | Low risk | No concerns | Some concerns | Major concerns | Major concerns | Very low |

# Network meta-analysis results

## 5.1.Balance ability


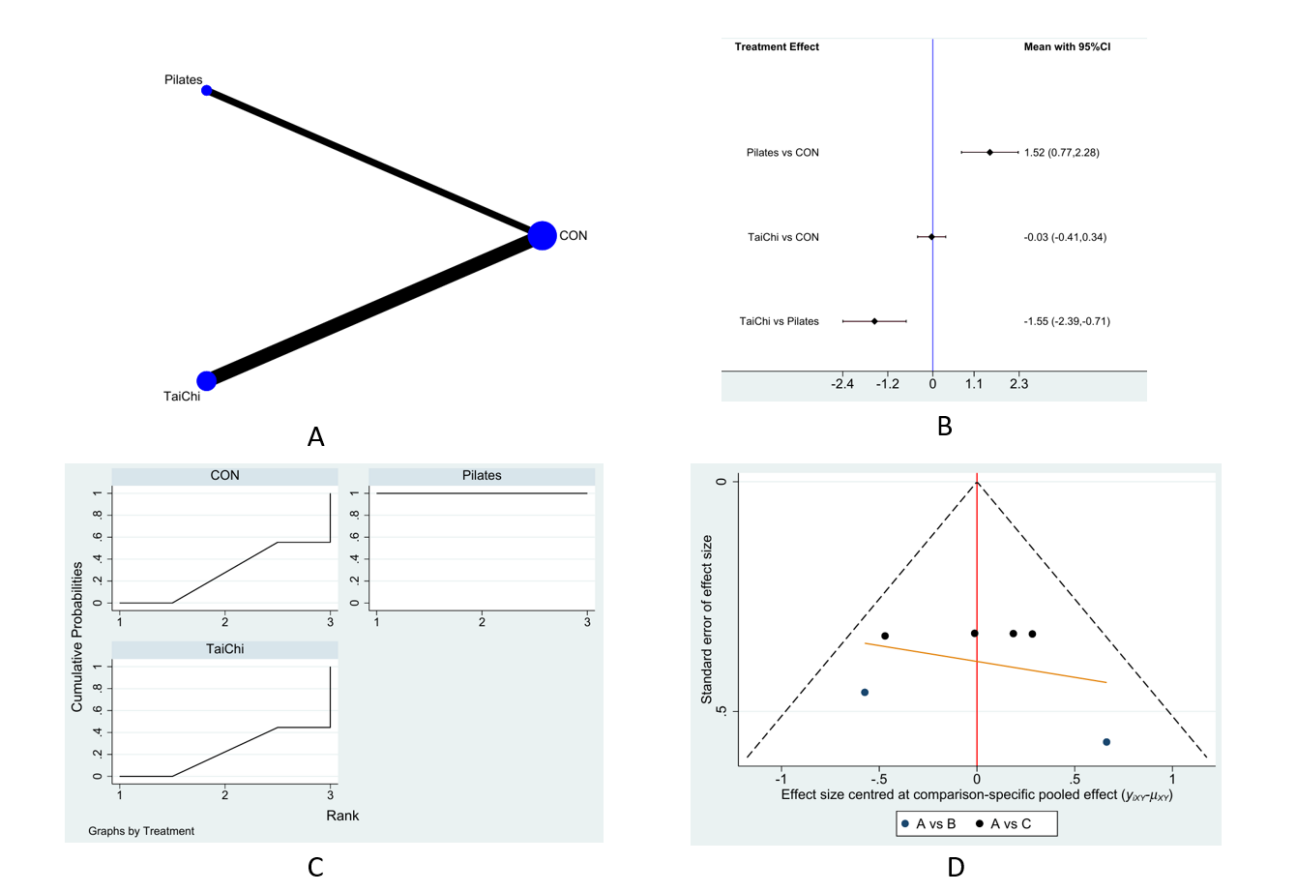


**A: Network map illustrating the geometry of direct treatment comparisons included in the network. Nodes represent different interventions, with node size proportional to the total sample size, and edges represent available direct evidence, with line thickness reflecting the number of contributing studies. Indirect comparisons were derived statistically through connected treatment pathways and are not displayed as separate edges in the network map.**

**B: Forest plot showing pairwise and network effect estimates expressed as standardized mean differences (SMDs) with 95% confidence intervals (CIs).**

**C: SUCRA ordination diagram displaying the cumulative ranking probabilities of each intervention.**

**D: Comparison-adjusted funnel plot assessing small-study effects and potential publication bias.**

## 5.2.Aerobic capacity


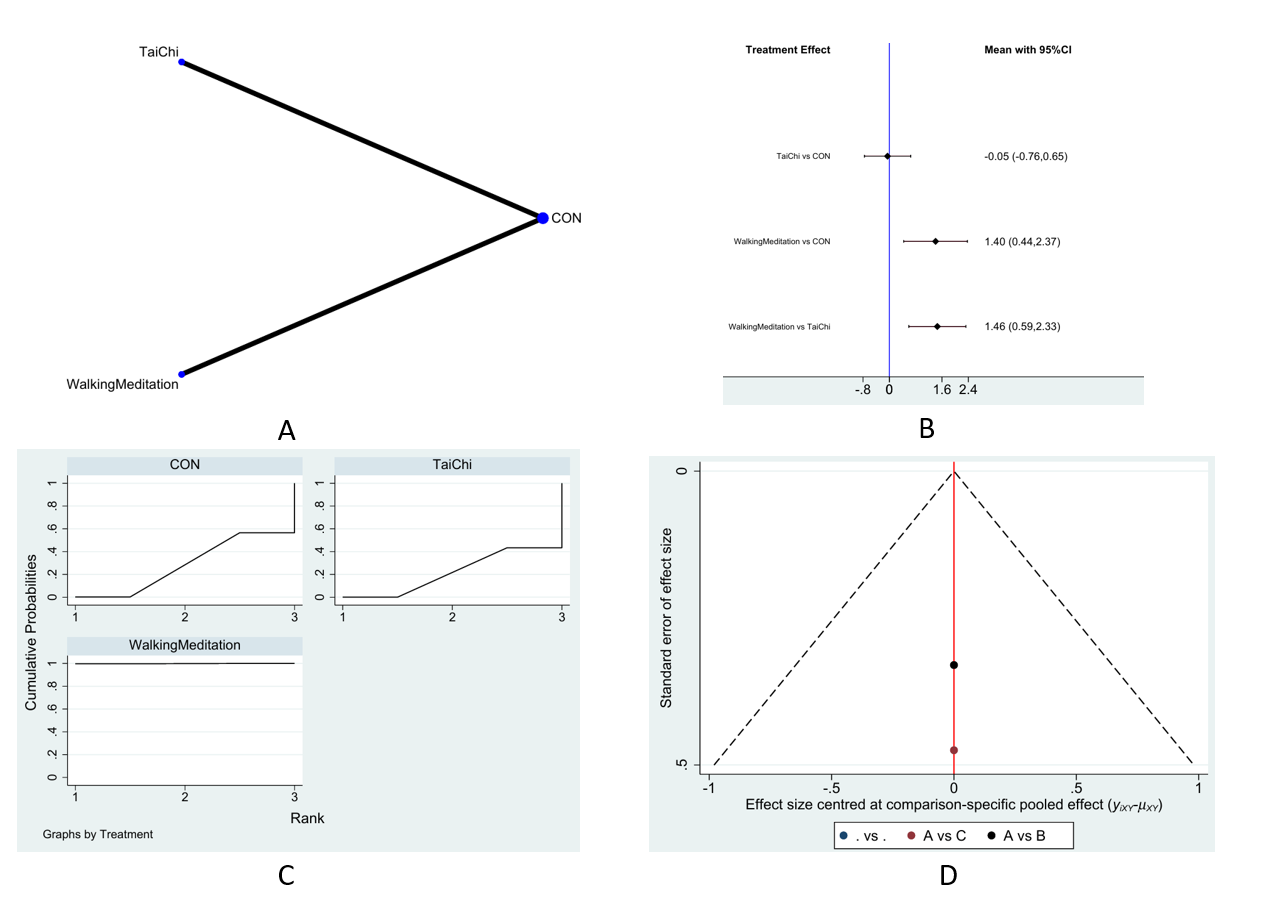


**A: Network map illustrating the geometry of direct treatment comparisons included in the network. Nodes represent different interventions, with node size proportional to the total sample size, and edges represent available direct evidence, with line thickness reflecting the number of contributing studies. Indirect comparisons were derived statistically through connected treatment pathways and are not displayed as separate edges in the network map.**

**B: Forest plot showing pairwise and network effect estimates expressed as standardized mean differences (SMDs) with 95% confidence intervals (CIs).**

**C: SUCRA ordination diagram displaying the cumulative ranking probabilities of each intervention.**

**D: Comparison-adjusted funnel plot assessing small-study effects and potential publication bias.**

## 5.3.Walking ability


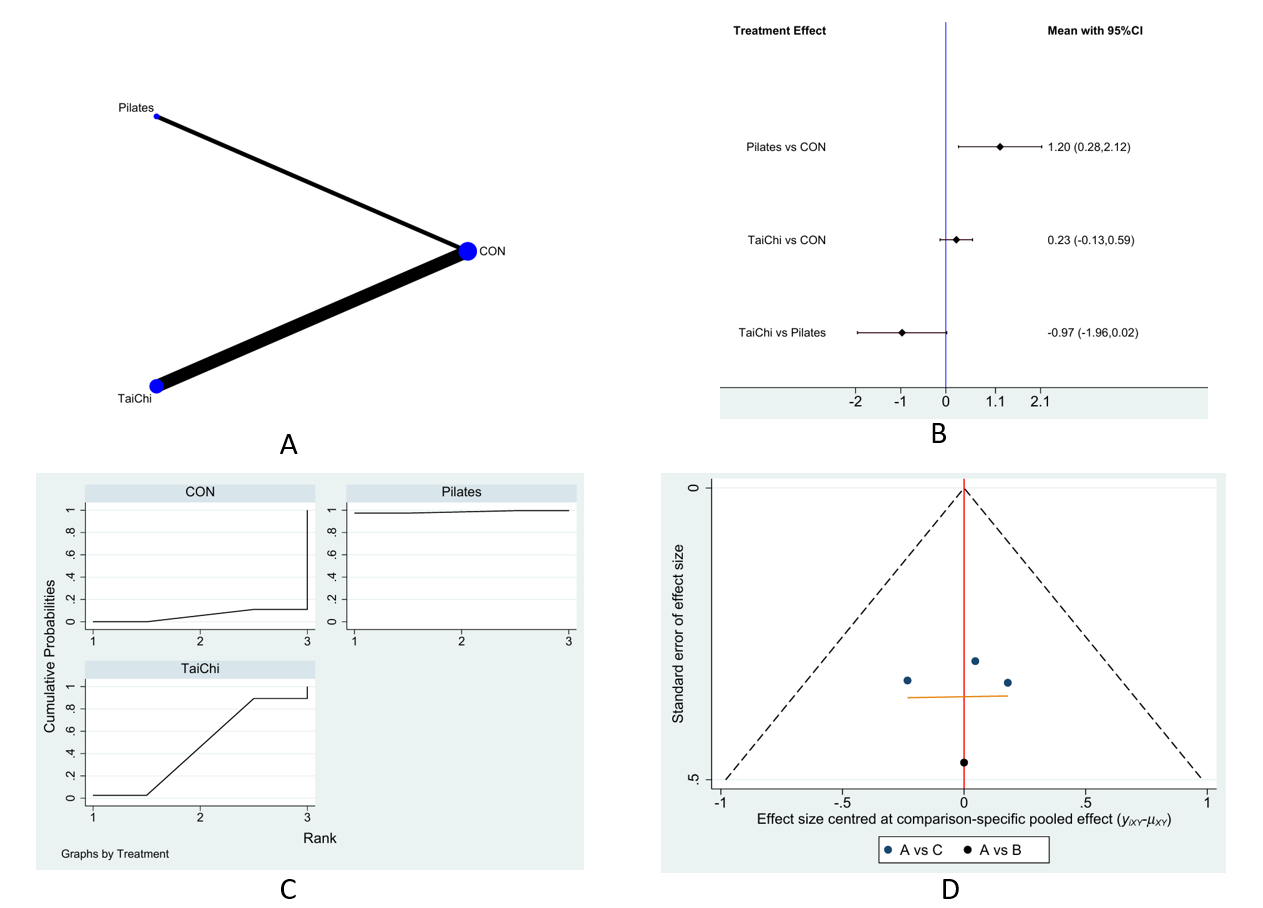


**A: Network map illustrating the geometry of direct treatment comparisons included in the network. Nodes represent different interventions, with node size proportional to the total sample size, and edges represent available direct evidence, with line thickness reflecting the number of contributing studies. Indirect comparisons were derived statistically through connected treatment pathways and are not displayed as separate edges in the network map.**

**B: Forest plot showing pairwise and network effect estimates expressed as standardized mean differences (SMDs) with 95% confidence intervals (CIs).**

**C: SUCRA ordination diagram displaying the cumulative ranking probabilities of each intervention.**

**D: Comparison-adjusted funnel plot assessing small-study effects and potential publication bias.**

## 5.4.Muscle strength


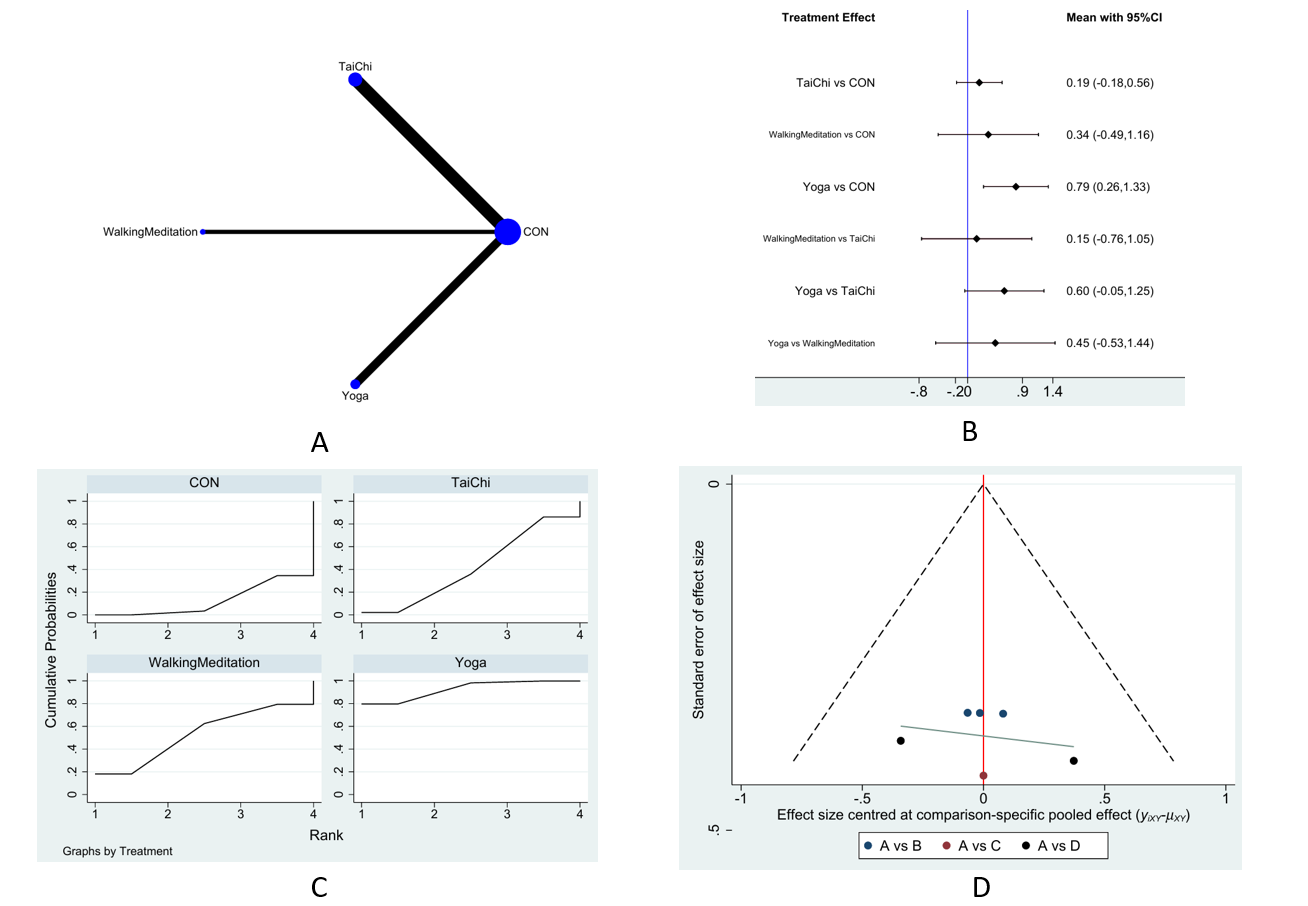


**A: Network map illustrating the geometry of direct treatment comparisons included in the network. Nodes represent different interventions, with node size proportional to the total sample size, and edges represent available direct evidence, with line thickness reflecting the number of contributing studies. Indirect comparisons were derived statistically through connected treatment pathways and are not displayed as separate edges in the network map.**

**B: Forest plot showing pairwise and network effect estimates expressed as standardized mean differences (SMDs) with 95% confidence intervals (CIs).**

**C: SUCRA ordination diagram displaying the cumulative ranking probabilities of each intervention.**

**D: Comparison-adjusted funnel plot assessing small-study effects and potential publication bias.**

## 5.5.Depression


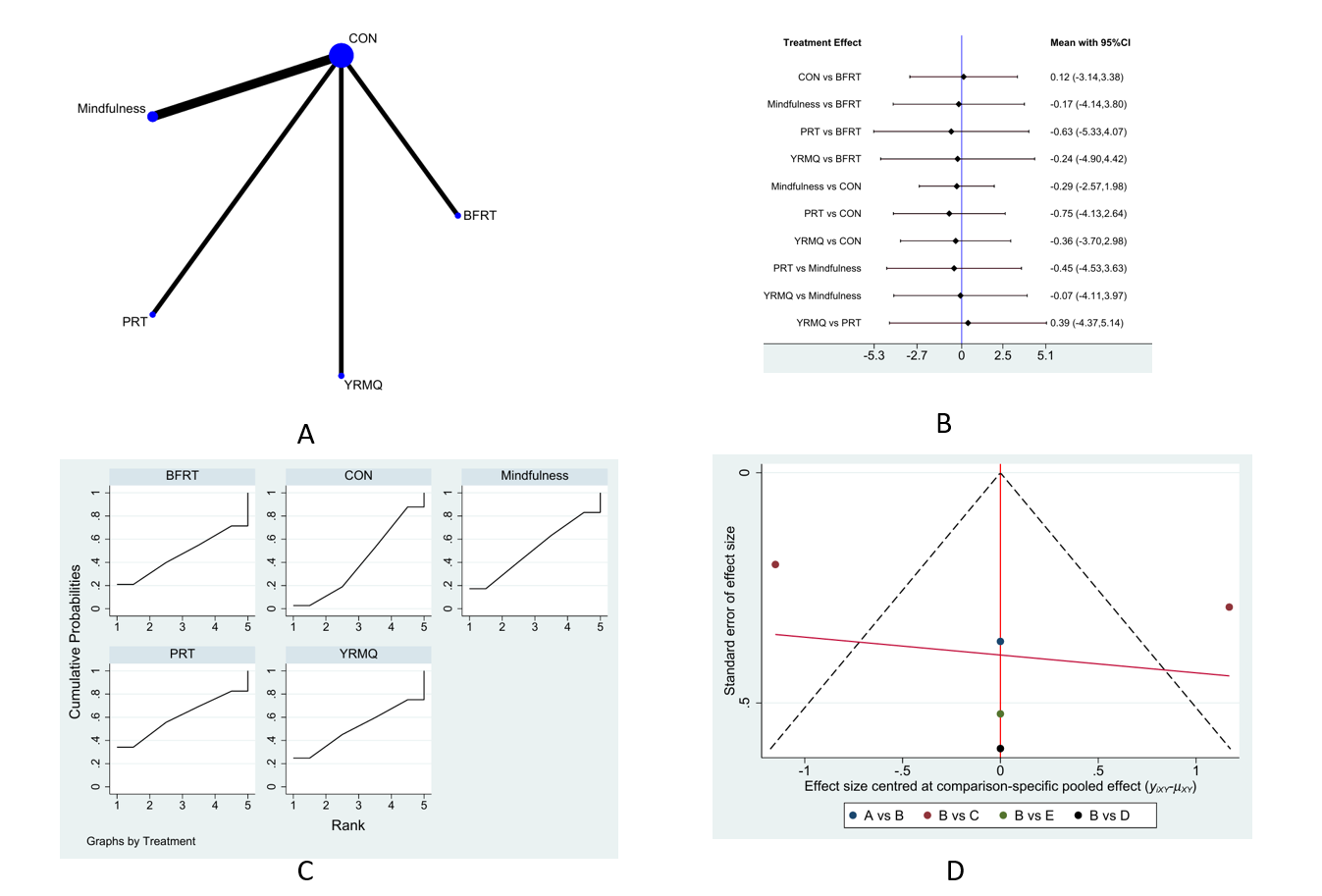


**A: Network map illustrating the geometry of direct treatment comparisons included in the network. Nodes represent different interventions, with node size proportional to the total sample size, and edges represent available direct evidence, with line thickness reflecting the number of contributing studies. Indirect comparisons were derived statistically through connected treatment pathways and are not displayed as separate edges in the network map.**

**B: Forest plot showing pairwise and network effect estimates expressed as standardized mean differences (SMDs) with 95% confidence intervals (CIs).**

**C: SUCRA ordination diagram displaying the cumulative ranking probabilities of each intervention.**

**D: Comparison-adjusted funnel plot assessing small-study effects and potential publication bias.**

## 5.6.Anxiety


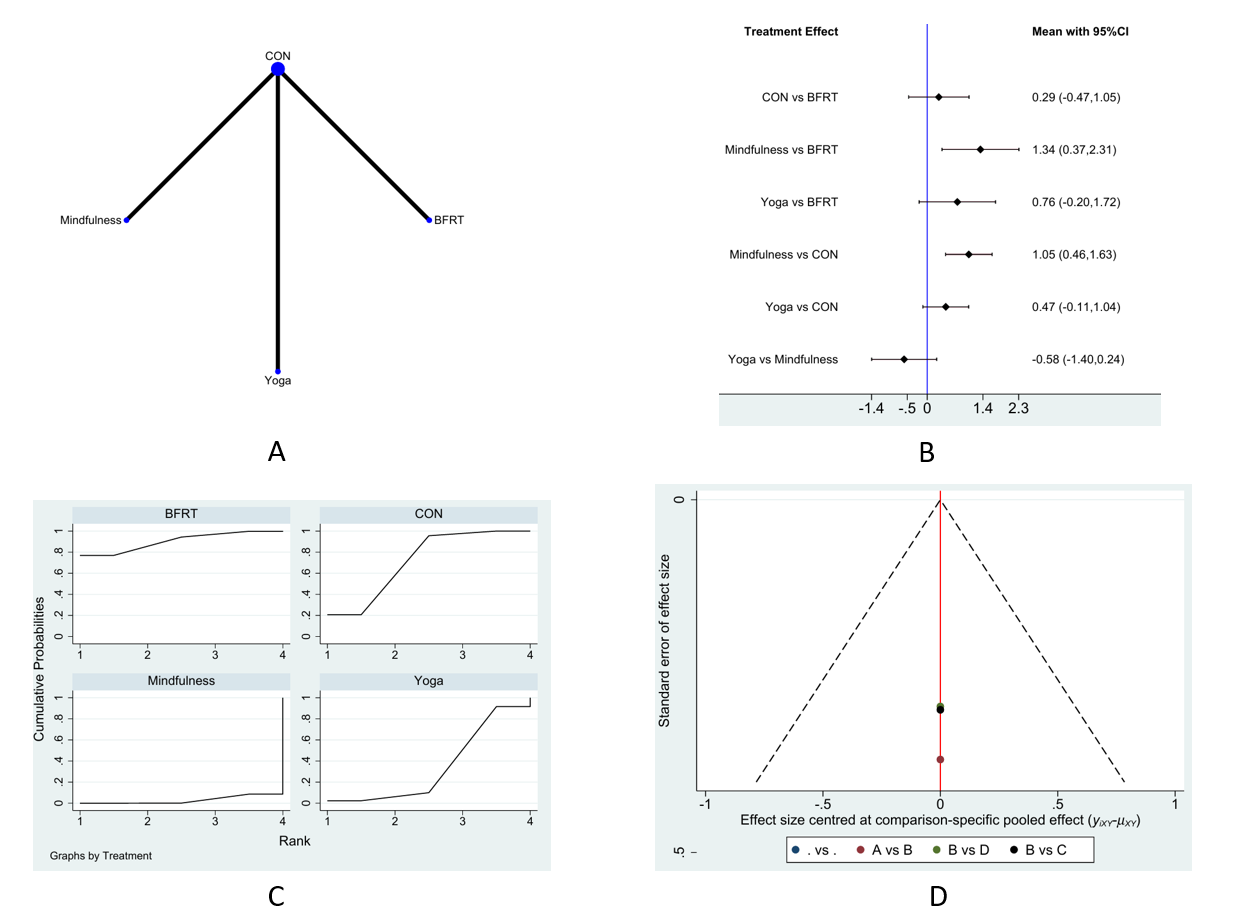


**A: Network map illustrating the geometry of direct treatment comparisons included in the network. Nodes represent different interventions, with node size proportional to the total sample size, and edges represent available direct evidence, with line thickness reflecting the number of contributing studies. Indirect comparisons were derived statistically through connected treatment pathways and are not displayed as separate edges in the network map.**

**B: Forest plot showing pairwise and network effect estimates expressed as standardized mean differences (SMDs) with 95% confidence intervals (CIs).**

**C: SUCRA ordination diagram displaying the cumulative ranking probabilities of each intervention.**

**D: Comparison-adjusted funnel plot assessing small-study effects and potential publication bias.**

## 5.7.Stress


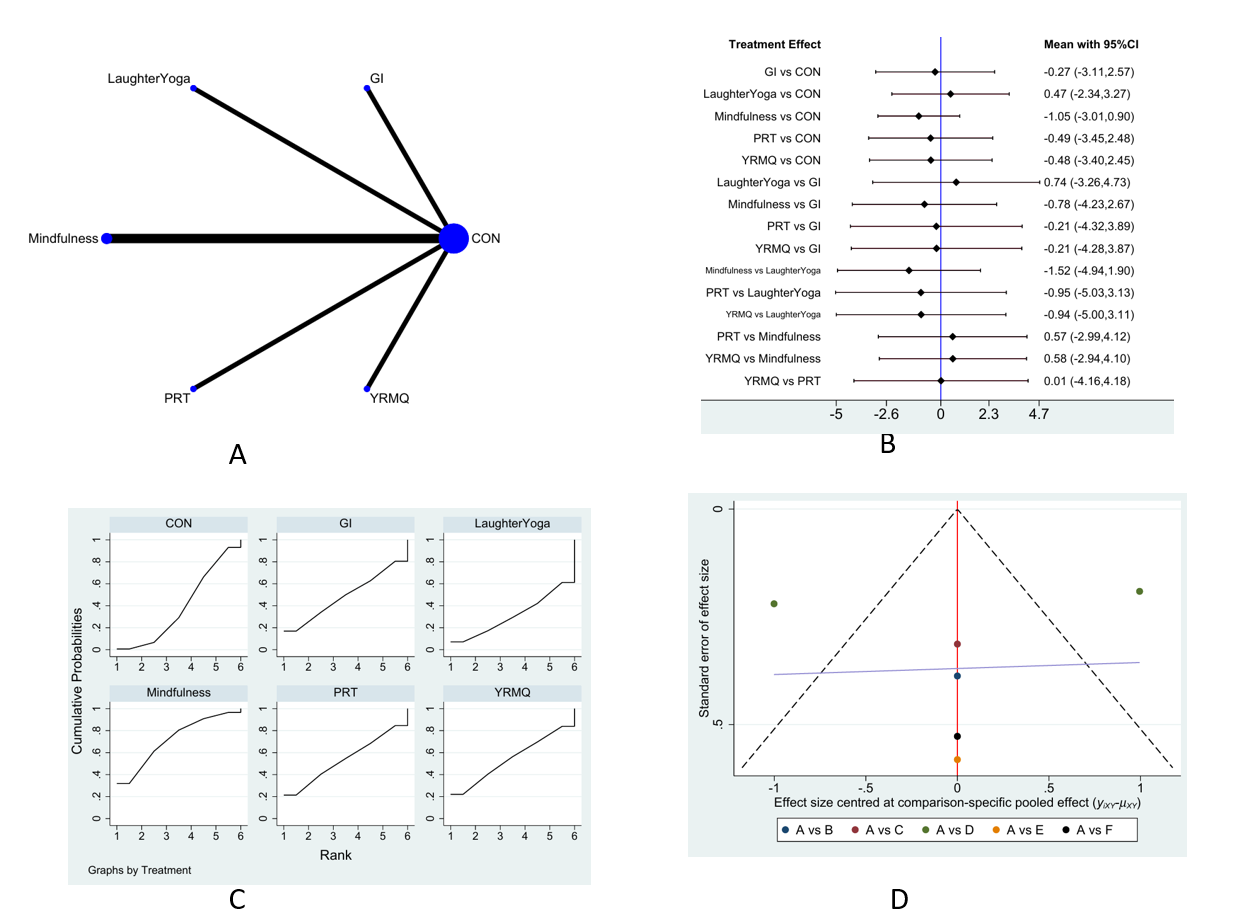


**A: Network map illustrating the geometry of direct treatment comparisons included in the network. Nodes represent different interventions, with node size proportional to the total sample size, and edges represent available direct evidence, with line thickness reflecting the number of contributing studies. Indirect comparisons were derived statistically through connected treatment pathways and are not displayed as separate edges in the network map.**

**B: Forest plot showing pairwise and network effect estimates expressed as standardized mean differences (SMDs) with 95% confidence intervals (CIs).**

**C: SUCRA ordination diagram displaying the cumulative ranking probabilities of each intervention.**

**D: Comparison-adjusted funnel plot assessing small-study effects and potential publication bias.**

# Leave-one-out method for sensitivity analysis

| Excluded_study | Mean | SE | Lower | Upper | CrI_Width |
| --- | --- | --- | --- | --- | --- |
| Balance ability |  |  |  |  |  |
| study 4 | 1.08 | 1.42 | -1.7 | 3.86 | 5.56 |
| study 40 | 0.96 | 1.38 | -1.75 | 3.67 | 5.42 |
| study 56 | 1.42 | 1.31 | -1.15 | 3.99 | 5.14 |
| study 560 | 0.87 | 1.36 | -1.79 | 3.53 | 5.32 |
| study 5600 | 1.15 | 1.34 | -1.48 | 3.78 | 5.26 |
| study 56000 | 1.1 | 1.33 | -1.51 | 3.71 | 5.22 |
| Aerobic capacity |  |  |  |  |  |
| study 16 | -5.7 | 35.61 | -75.5 | 64.1 | 139.6 |
| study 56 | 2.7 | 0.79 | 1.15 | 4.25 | 3.1 |
| Walking ability |  |  |  |  |  |
| study 4 | 0.18 | 0.29 | -0.39 | 0.75 | 1.14 |
| study 33 | 0.97 | 0.44 | 0.11 | 1.83 | 1.72 |
| study 56 | 0.71 | 0.41 | -0.09 | 1.51 | 1.6 |
| study 560 | 0.73 | 0.42 | -0.09 | 1.55 | 1.64 |
| Muscle strength |  |  |  |  |  |
| study 13 | 2.41 | 1.89 | -1.29 | 6.11 | 7.4 |
| study 130 | 3.02 | 1.97 | -0.85 | 6.89 | 7.74 |
| study 16 | 3.28 | 2.05 | -0.74 | 7.3 | 8.04 |
| study 56 | 3.71 | 2.18 | -0.57 | 7.99 | 8.56 |
| study 560 | 3.44 | 2.11 | -0.7 | 7.58 | 8.28 |
| study 5600 | 3.09 | 2.02 | -0.87 | 7.05 | 7.92 |
| Depression |  |  |  |  |  |
| study 9 | -0.62 | 1.29 | -3.14 | 1.9 | 5.04 |
| study 39 | -6.34 | 4.34 | -14.85 | 2.16 | 17.02 |
| study 42 | -7.13 | 4.96 | -16.86 | 2.59 | 19.45 |
| study 47 | -6.11 | 4.39 | -14.71 | 2.5 | 17.21 |
| study 470 | -6.05 | 5.41 | -16.65 | 4.56 | 21.21 |
| Anxiety |  |  |  |  |  |
| study 5 | -3.02 | 2.11 | -7.16 | 1.12 | 8.28 |
| study 39 | -7.01 | 3.62 | -14.1 | 0.08 | 14.18 |
| study 42 | -5.42 | 4.08 | -13.41 | 2.57 | 15.98 |
| Stress |  |  |  |  |  |
| study 9 | -1.92 | 1.34 | -4.55 | 0.71 | 5.26 |
| study 23 | -6.84 | 3.11 | -12.94 | -0.74 | 12.2 |
| study 32 | -6.11 | 3.45 | -12.87 | 0.65 | 13.52 |
| study 47 | -5.48 | 3.21 | -11.77 | 0.81 | 12.58 |
| study 470 | -5.36 | 3.39 | -12.01 | 1.29 | 13.3 |
| study 60 | -5.72 | 3.28 | -12.15 | 0.71 | 12.86 |

Leave-one-out sensitivity analyses revealed heterogeneity in the robustness of the pooled effects across outcomes. For muscle strength and balance, the pooled estimates remained stable in both magnitude and direction after exclusion of any individual study, indicating that these results were not driven by single trials. In contrast, the pooled effects for depression and stress were sensitive to the exclusion of specific influential studies, particularly study 9, whose removal substantially attenuated the overall effect and rendered the estimates non-significant. For stress, exclusion of study 23 also resulted in a noticeable change in the magnitude and statistical significance of the pooled effect. Anxiety and mobility outcomes showed generally consistent effect directions across analyses; however, the precision of the pooled estimates was influenced by individual studies with large within-group variability, most notably study 39 for anxiety and study 33 for mobility. For aerobic capacity, sensitivity analyses demonstrated extreme instability, as only two trials were available; exclusion of either study resulted in pooled estimates entirely driven by the remaining single trial. Overall, these findings indicate that while some outcomes were robust, others were sensitive to the influence of specific studies and should be interpreted with caution.
